# Investigation of Fenebrutinib Metabolism and Bioactivation Using MS^3^ Methodology in Ion Trap LC/MS

**DOI:** 10.3390/molecules28104225

**Published:** 2023-05-22

**Authors:** Aishah M. Alsibaee, Haya I. Aljohar, Mohamed W. Attwa, Ali S. Abdelhameed, Adnan A. Kadi

**Affiliations:** Department of Pharmaceutical Chemistry, College of Pharmacy, King Saud University, Riyadh 11451, Saudi Arabia

**Keywords:** fenebrutinib, Bruton tyrosine kinase inhibitor, metabolism, reactive intermediates, trapping agents, LC-ITMS

## Abstract

Fenebrutinib is an orally available Bruton tyrosine kinase inhibitor. It is currently in multiple phase III clinical trials for the management of B-cell tumors and autoimmune disorders. Elementary in-silico studies were first performed to predict susceptible sites of metabolism and structural alerts for toxicities by StarDrop WhichP450™ module and DEREK software; respectively. Fenebrutinib metabolites and adducts were characterized in-vitro in rat liver microsomes (RLM) using MS3 method in Ion Trap LC-MS/MS. Formation of reactive and unstable intermediates was explored using potassium cyanide (KCN), glutathione (GSH) and methoxylamine as trapping nucleophiles to capture the transient and unstable iminium, 6-iminopyridin-3(*6H*)-one and aldehyde intermediates, respectively, to generate a stable adducts that can be investigated and analyzed using mass spectrometry. Ten phase I metabolites, four cyanide adducts, five GSH adducts and six methoxylamine adducts of fenebrutinib were identified. The proposed metabolic reactions involved in formation of these metabolites are hydroxylation, oxidation of primary alcohol to aldehyde, n-oxidation, and n-dealkylation. The mechanism of reactive intermediate formation of fenebrutinib can provide a justification of the cause of its adverse effects. Formation of iminium, iminoquinone and aldehyde intermediates of fenebrutinib was characterized. N-dealkylation followed by hydroxylation of the piperazine ring is proposed to cause the bioactivation to iminium intermediates captured by cyanide. Oxidation of the hydroxymethyl group on the pyridine moiety is proposed to cause the generation of reactive aldehyde intermediates captures by methoxylamine. N-dealkylation and hydroxylation of the pyridine ring is proposed to cause formation of iminoquinone reactive intermediates captured by glutathione. FBB and several phase I metabolites are bioactivated to fifteen reactive intermediates which might be the cause of adverse effects. In the future, drug discovery experiments utilizing this information could be performed, permitting the synthesis of new drugs with better safety profile. Overall, in silico software and in vitro metabolic incubation experiments were able to characterize the FBB metabolites and reactive intermediates using the multistep fragmentation capability of ion trap mass spectrometry.

## 1. Introduction

**Figure 1:**
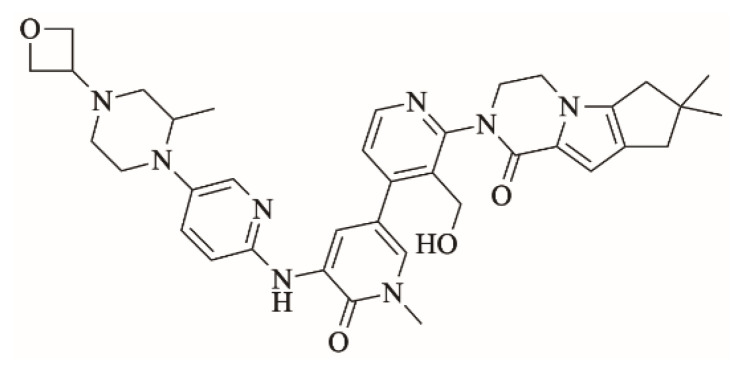
Chemical structure of fenebrutinib.

Fenebrutinib (Figure 1; FBB) is an investigational orally available Bruton tyrosine kinase inhibitor developed by Roche pharmaceuticals [1]. FBB inhibits B cell signaling pathways. It is used for the management of B cell tumors and autoimmune disorders specifically rheumatoid arthritis and multiple sclerosis [2,3]. FBB shows the highest potency of Bruton tyrosine kinase inhibitors in phase III clinical trials for multiple sclerosis [4,5]. Adverse effects of FBB include: nausea, vomiting, bleeding, bruising, alanine aminotransferase elevation [6].

Drug metabolism studies are crucial for investigational drugs. Identification of drug metabolites and their possible toxicities is very important [7,8]. Rat liver microsomes are used to investigate drug metabolism in vitro [9]. LC/MS is an efficient technology to identify and characterize drug metabolites [10,11,12,13]. Most drugs are transformed into polar and stable metabolites that aid into their excretion from the body. However, some drugs are bioactivated into unstable electrophiles that has the ability to bind and damage DNA and proteins [14,15]. The formation of such reactive intermediates can be investigated in vitro using RLMs and trapping agents. So as to identify compounds with probable toxicity problems, definite care is set to structural alerts, that have the ability to generate reactive molecular intermediates via metabolism bioactivation. The awareness of such information is necessary in order to decrease the probability that new candidates (pharmaceuticals) might have toxic side effects. Characterization of reactive intermediates is crucial step in the procedure of new drug design with expected good toxicological features [16,17]. Liquid chromatography mass spectrometry (LC-MS) is deliberated the analytical tool of choice that could be used for characterization of reactive intermediates [18,19,20]. The unstable and transient nature of reactive intermediates makes it is difficult to be detected directly. Instead, a trapping agent for reactive intermediates resulted in adducts formation of that are stable and can be detected and characterized by LC-MS [21]. Trapping agents binds to the unstable reactive intermediates and make it easier for investigators to characterize and identify these intermediates using mass spectrometry [22,23,24,25]. In silico toxicity evaluation of the FBB and its metabolites were performed using the DEREK module of software, while the XenoSite and StarDrop software were used to prove the bioactivation theory [26,27].

## 2. Results and Discussion

### 2.1. Results of In Silico FBB Metabolism and Toxicity Prediction

**Figure 2:**
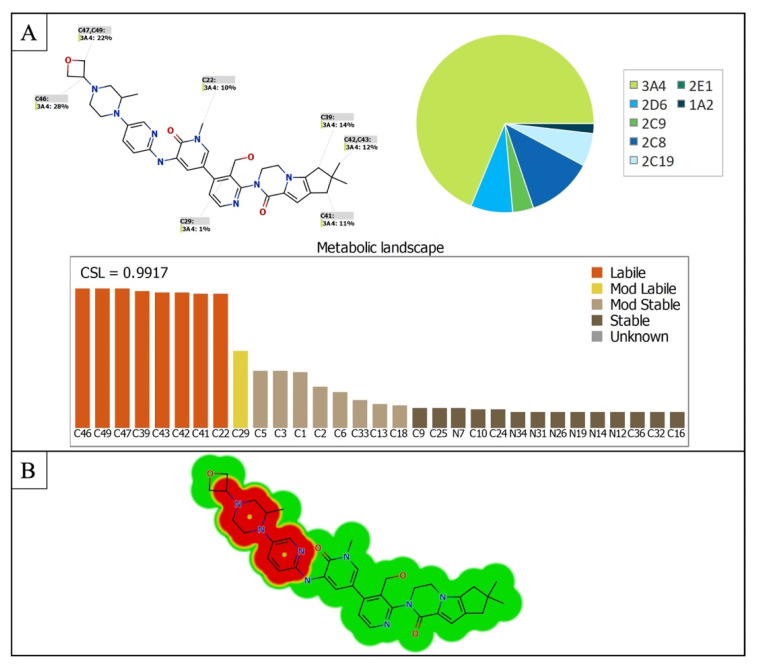
(**A**) Predicted metabolic sites for FBB by StarDrop WhichP450 module. (**B**) Structural alerts of spebrutinib by DEREK module of StarDrop software.

StarDrop software predictions of FBB metabolism indicates the lability of each site with respect to metabolism by different isoforms of CYP450 enzymes. This indicates that C46, C47 and C49 in the oxetane ring, C22 the methyl group on the nitrogen atom in the pyridine ring, C39-43 at the other end of the molecule are predicted to be labile to metabolism by CYP450 enzymes especially CYP3A4. The composite site liability (CSL) is shown in the top-right of the metabolic landscape (Figure 2A) and its value is 0.9917 which predicts that FBB is highly susceptible to metabolism by CYP450 isoforms. The results of the WhichP450™ module, concluded in the pie chart is predicting the most likely CYP450 isoforms that has a critical role in FBB metabolism (Figure 2A). According to these results, CYP3A4 isoform has the major role in FBB metabolism [28,29,30].

DEREK software was used to assess the possible toxicities of FBB based on its chemical structure. FBB shows possibility of causing HERG channel inhibition (plausible) due to the piperazine moiety and the adjacent pyridine ring (highlighted in red in Figure 2B) [31].

### 2.2. Fragment Ions Study of FBB

**Figure 3:**
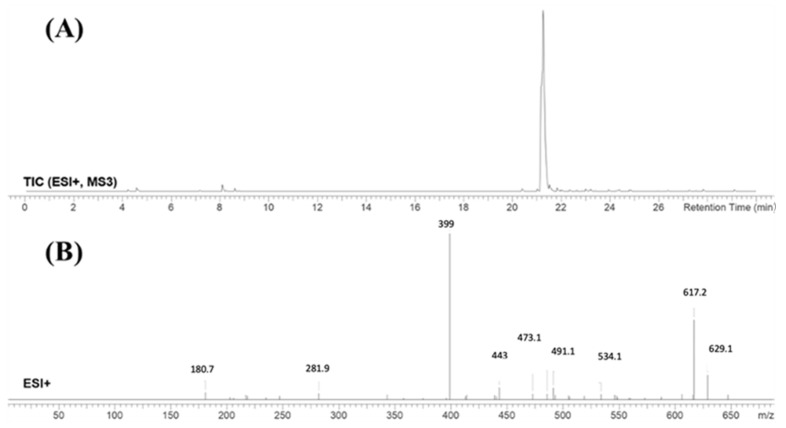
Product ion chromatogram of FBB (**A**). Product ion mass spectrum of FBB (**B**).

**Scheme 1:**
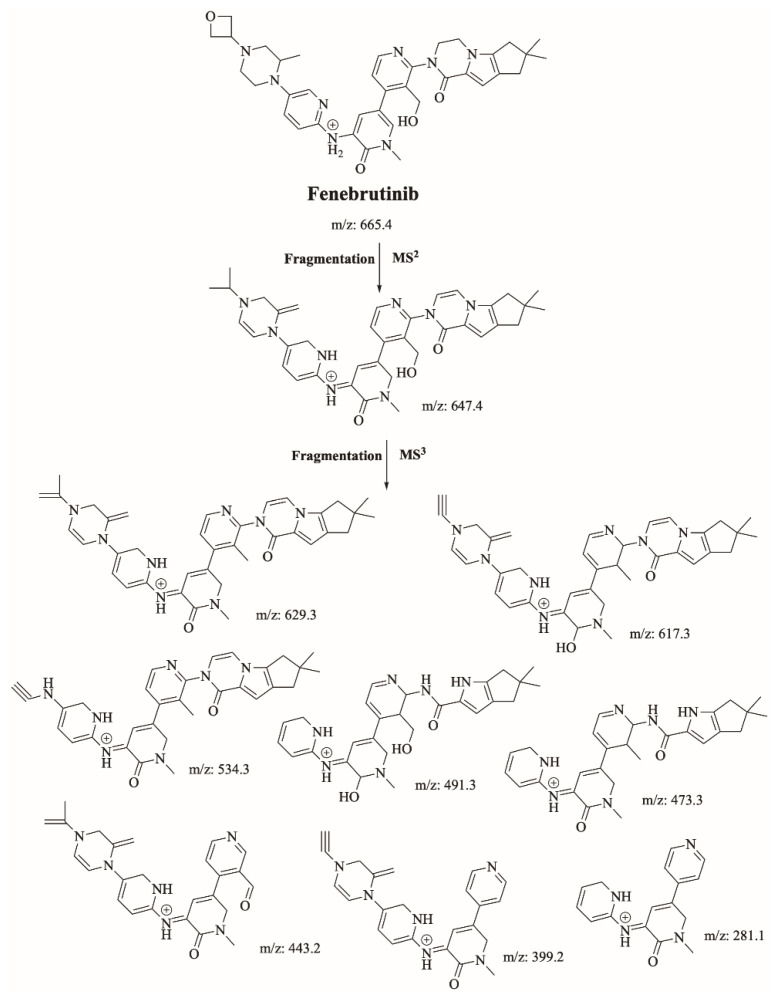
Proposed interpretation of FBB fragmentation.

FBB peak elutes at 21.2 min in product ion chromatogram (Figure 3). Dissociation of FBB ion at *m*/*z* 665 inside the collision cell produces one fragment at *m*/*z* 647 (loss of water molecule). Further investigation using MS^3^ analysis of fragment *m*/*z* 647 yielded eight characteristic and qualitative fragment ions at *m*/*z* 629.1, *m*/*z* 617.2, *m*/*z* 534.1, *m*/*z* 491.1, *m*/*z* 473.1, *m*/*z* 443, *m*/*z* 399 and *m*/*z* 281.9 (Scheme 1).

### 2.3. Identification of FBB Related Metabolites

**Figure 4:**
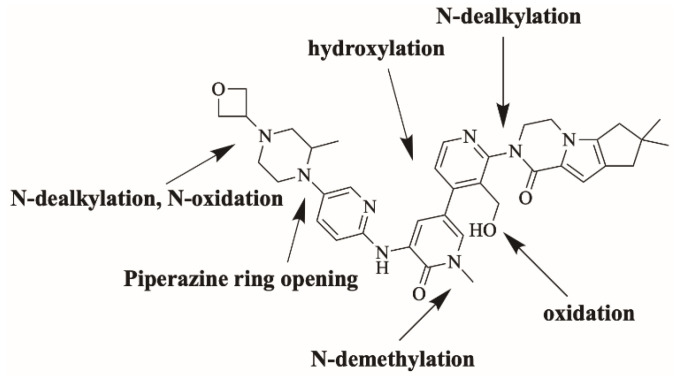
Proposed metabolic pathways of FBB.

**Figure 5:**
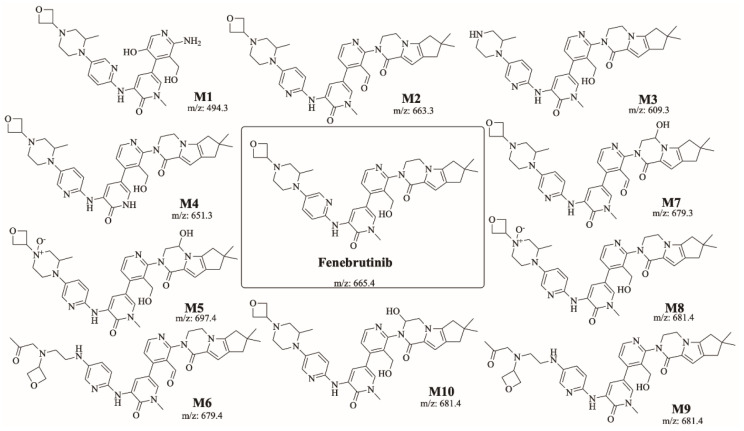
Summary of the ten proposed metabolites of FBB.

FBB incubation in RLM resulted in the characterization of ten in vitro metabolites. Proposed reactions by CYP450 enzymes include hydroxylation, oxidation, N-dealkylation and N-oxidation. Different sites of the drug are susceptible to metabolism by the most vulnerable site is the piperazine ring (Figure 4). Figure 5 shows chemical structures of proposed metabolites. The details for all other FBB metabolites are exhibited in the supplementary file (Supplementary Materials: Figures S1–S9; Table S1).

#### Identification of M1

**Figure 6:**
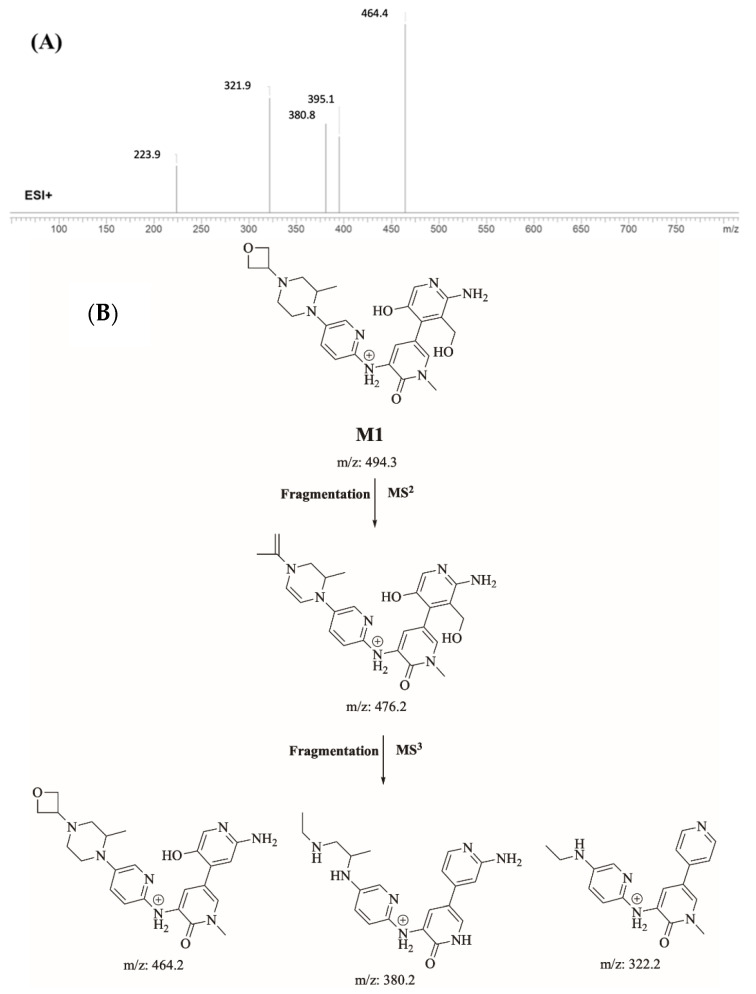
Product ion mass spectrum of M1 (**A**). Proposed interpretation of fragmentation of M1 (**B**).

M1 (*m*/*z* 494.3) is proposed to be generated by hydroxylation and N-dealkylation of FBB. M1 peak elutes at 18.4 min in product ion chromatogram. Dissociation of FBB ion at *m*/*z* 494.3 inside the collision cell produces one fragment at *m*/*z* 476 (loss of water molecule). Further investigation using MS^3^ analysis of fragment *m*/*z* 476 yielded three characteristic fragment ions at *m*/*z* 464.4, *m*/*z* 380.8 and *m*/*z* 321.9 (Figure 6).

### 2.4. Identification of Iminium Reactive Intermediates Using Potassium Cyanide as Trapping Agent

#### 2.4.1. Identification of M11/KCN Cyanide Adduct

**Figure 7:**
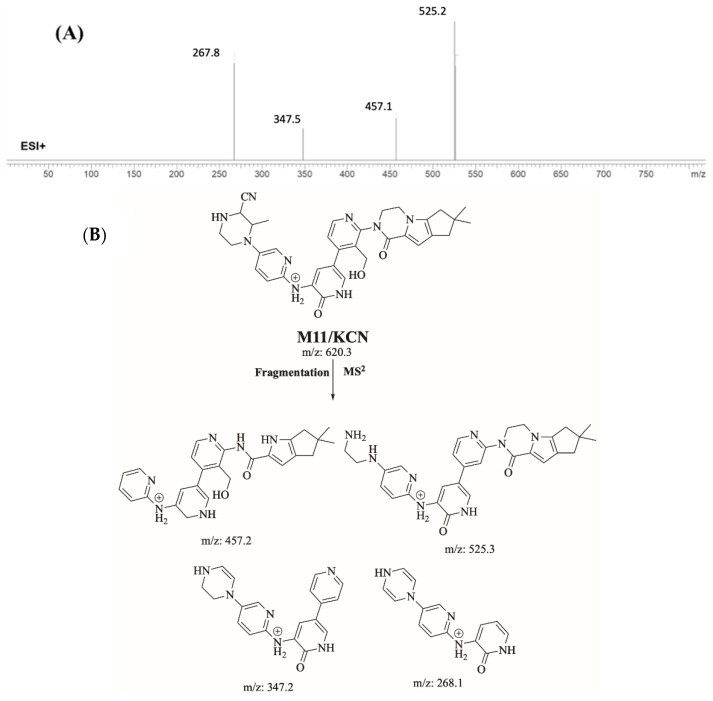
Product ion mass spectrum of M11/KCN cyanide adduct (**A**) and proposed interpretation of fragmentation of M11/KCN (**B**).

M11/KCN is proposed to form by the addition of cyanide group to M3 of FBB. M11/KCN (*m*/*z* 620.3) peak appeared at 16.94 minute in product ion chromatogram. Dissociation of M11/KCN ion inside the collision cell produces four fragment ions at *m*/*z* 525.2 *m*/*z* 457.1, *m*/*z* 347.5 *m*/*z* and *m*/*z* 267.8 (Figure 7).

#### 2.4.2. Proposed Bioactivation Mechanism of FBB to Iminium Reactive Intermediates

**Figure 8:**
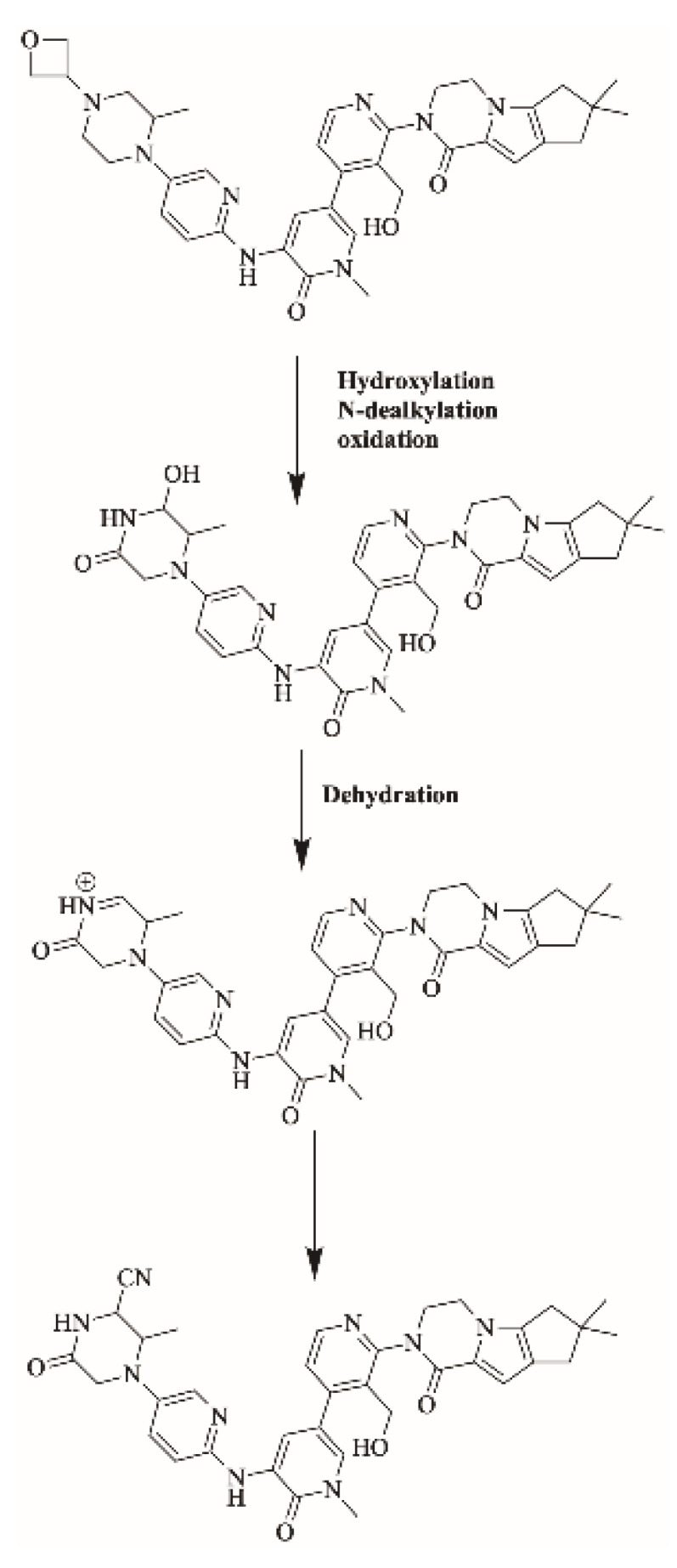
Proposed bioactivation pathways of FBB to iminium reactive intermediates trapped by potassium cyanide.

**Figure 9:**
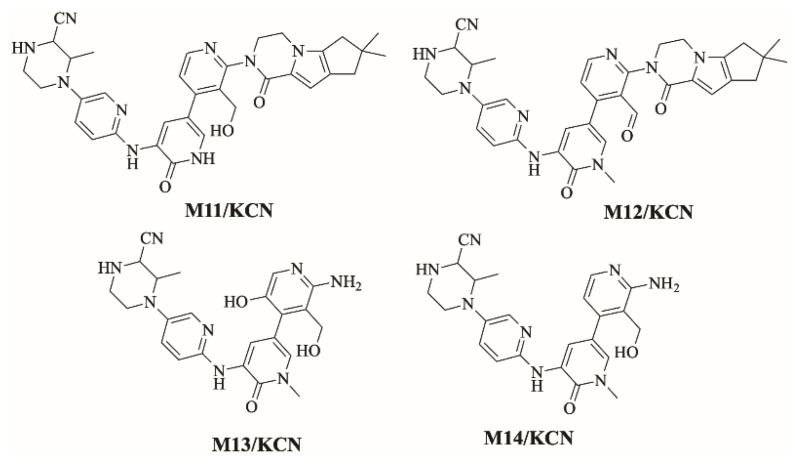
Summary of proposed cyanide adducts of FBB.

Figure 8 shows the bioactivation pathway for FBB to iminium intermediates. The generation of M11/KCN, M12/KCN, M13/KCN and M14/KCN cyanide adducts revealed the formation of iminium unstable intermediates in the piperazine ring during in vitro metabolism of FBB. Hydroxylation by CYP450 enzymes at piperazine moiety in FBB followed by loss of one water molecule (dehydration) lead to the formation of iminium ions intermediate which are unstable species that can be captured by cyanide as nucleophile forming stable adduct that can be characterized in mass spectrometry. Figure 9 summarizes the proposed cyanide adducts of FBB. The details for all other FBB cyano adducts are exhibited in the supplementary file (Supplementary Materials: Figures S10–S12; Table S2).

### 2.5. Identification of 6-Iminopyridin-3(6H)-One Reactive Intermediates Using Glutathione (GSH) as Trapping Agent

#### 2.5.1. Identification of M15/GSH GSH Adduct

**Figure 10:**
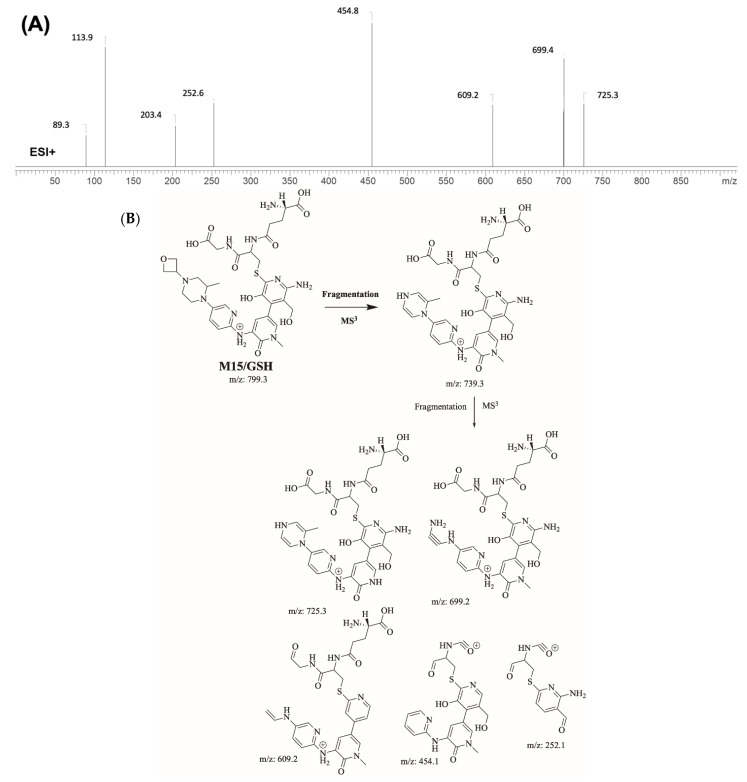
Product ion mass spectrum of M15/GSH adduct (**A**) and proposed interpretation of fragmentation of M15/GSH (**B**).

M15/GSH is proposed to form by the addition of GSH to the 6-iminopyridin-3(*6H*)-one reactive metabolite of M1 of FBB. M15/GSH (*m*/*z* 799.3) peak appeared at 17.3 min in fragment ion chromatogram. Dissociation of M15/GSH ion at *m*/*z* 799 inside the collision cell produces one fragment at *m*/*z* 739 (loss of trimethylene oxide ring). Further investigation using MS^3^ analysis of fragment *m*/*z* 739 yielded five characteristic and qualitative fragment ions at *m*/*z* 725.3, *m*/*z* 699.4, *m*/*z* 609.2 *m*/*z* 454.8 and *m*/*z* 252.6 (Figure 10).

#### 2.5.2. Proposed Bioactivation Mechanism of FBB to 6-Iminopyridin-3(*6H*)-One Reactive Intermediates

**Figure 11:**
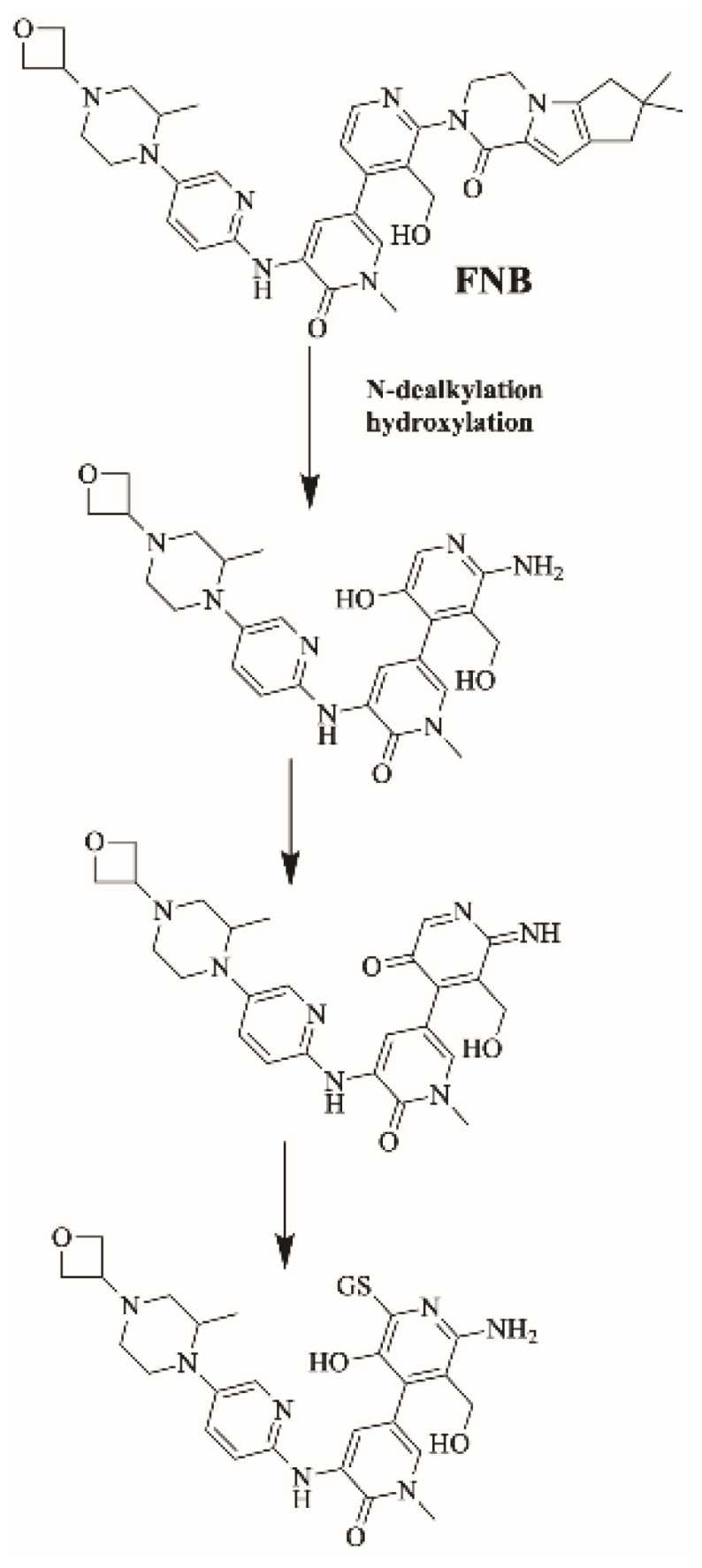
Proposed bioactivation pathways of FBB to 6-iminopyridin-3(*6H*)-one reactive intermediates trapped by GSH.

**Figure 12:**
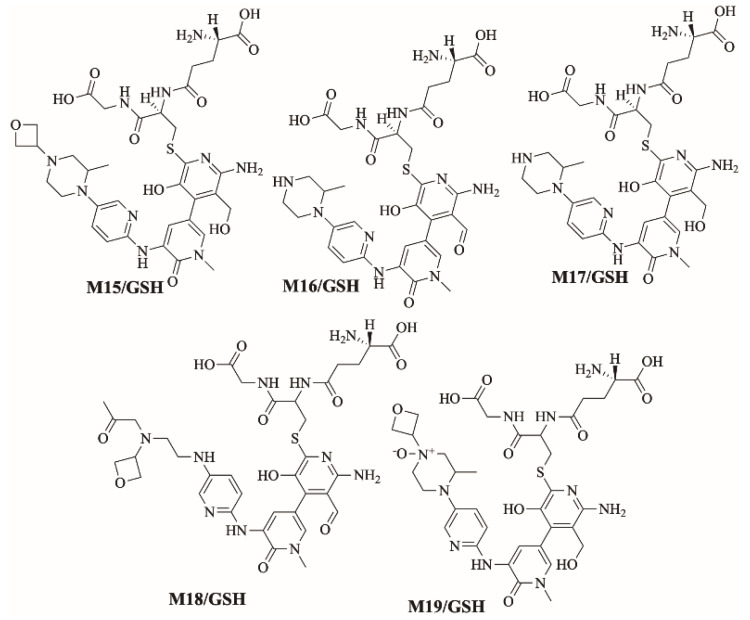
Summary of proposed GSH adducts of FBB.

Figure 11 shows the bioactivation pathway for FBB to 6-iminopyridin-3(*6H*)-one intermediates. N-dealkylation and hydroxylation of pyridine ring performed by CYP450 enzymes results in the formation of reactive 6-iminopyridin-3(*6H*)-one intermediates captured by glutathione. Figure 12 summarizes the proposed GSH adducts of FBB. The details for all other FBB GSH adducts are exhibited in the supplementary file (Supplementary Materials: Figures S13–S16; Table S3).

### 2.6. Identification of Aldehyde Reactive Intermediates Using Methoxylamine as Trapping Agent

#### 2.6.1. Identification of M20/CH_3_ONH_2_ Methoxylamine Adduct

**Figure 13:**
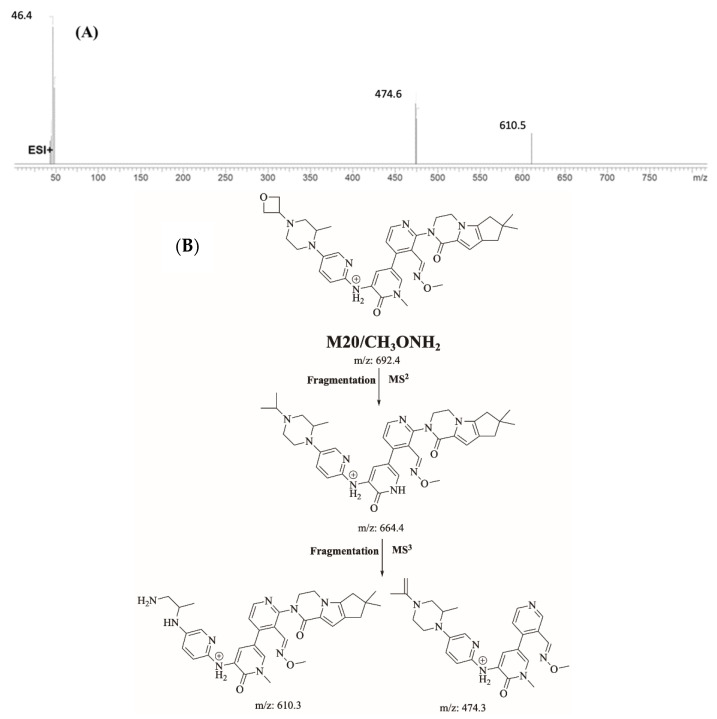
Product ion mass spectrum of M20/CH_3_ONH_2_ adduct (**A**) and proposed interpretation of fragmentation of M20/CH_3_ONH_2_ (**B**).

M20/CH_3_ONH_2_ is proposed to form by the addition of methoxylamine to aldehyde reactive intermediate of FBB. M20/CH_3_ONH_2_ (*m*/*z* 692.4) peak appeared at 22.4 min in fragment ion chromatogram. Dissociation of M20/CH_3_ONH_2_ ion at *m*/*z* 692.4 inside the collision cell produces one fragment at *m*/*z* 664.4. Further investigation using MS^3^ analysis of fragment *m*/*z* 664.4 yielded two characteristic and qualitative fragment ions at *m*/*z* 610.5 and *m*/*z* 474.6 (Figure 13).

#### 2.6.2. Proposed Bioactivation Mechanism of FBB to Aldehyde Reactive Intermediates

**Figure 14:**
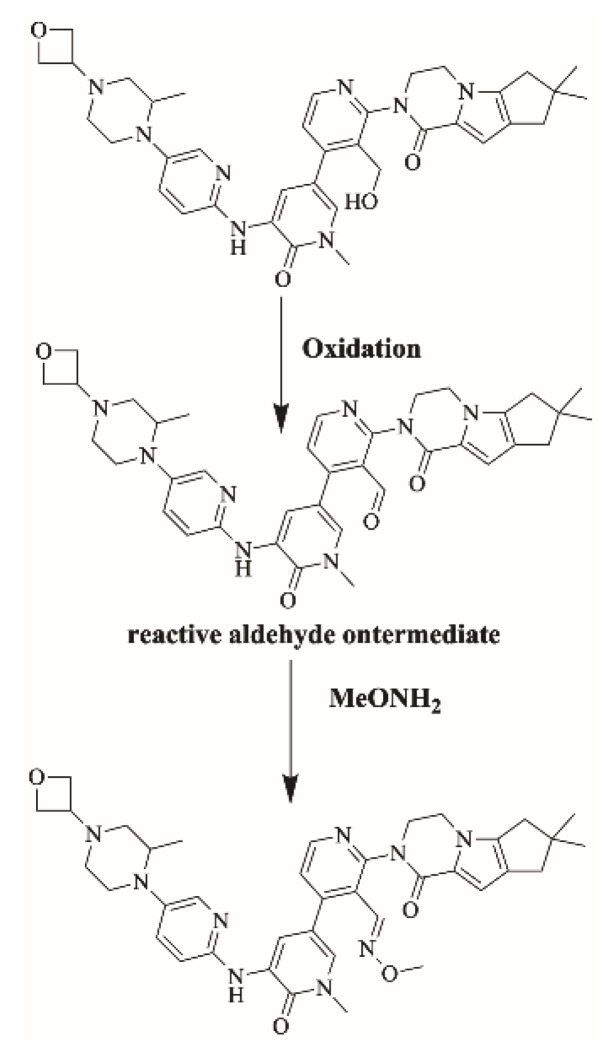
Proposed bioactivation pathways of FBB to aldehyde reactive intermediates trapped by methoxylamine.

**Figure 15:**
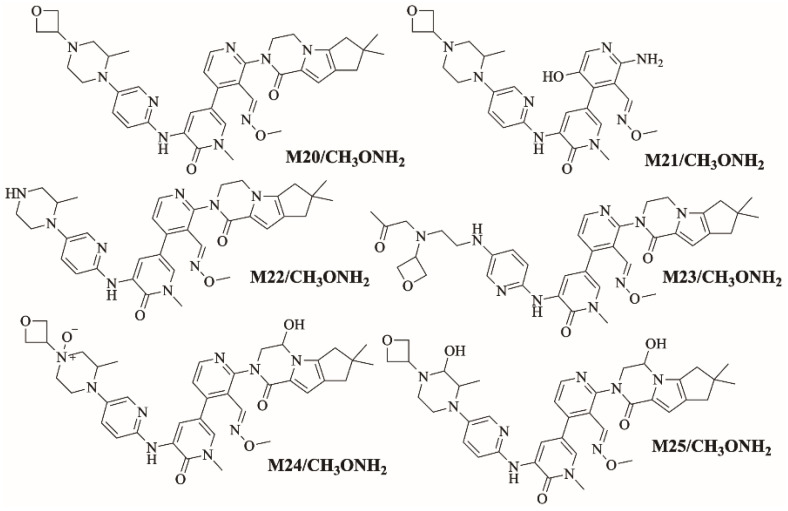
Summary of proposed methoxylamine adducts of FBB.

Figure 14 shows the bioactivation pathway for FBB to aldehyde intermediates. The generation of FBB692, FBB521, FBB636, FBB708 and FBB724a/b methoxylamine adducts (Figure 15) revealed the generation of aldehyde intermediates in the acrylamide group during in vitro metabolism of FBB [32]. Oxidation of primary alcohol by CYP450 enzymes leads to the formation of aldehyde reactive intermediates which are trapped by methoxylamine forming stable methoxylamine adducts that can be analyzed and detected in mass spectrometry [33]. The details for all other FBB methoxylamine adducts are exhibited in the supplementary file (Supplementary Materials: Figures S17–S21; Table S4).

## 3. Chemicals and Methods

### 3.1. Chemicals and Animals

Sprague Dawley rats were used for RLMs preparation [25,34]. Acetonitrile, formic acid, potassium cyanide, glutathione and methoxylamine were obtained from Sigma-Aldrich company (St. Louis, MO, USA). FBB was purchased from MedChemExpress company (Princeton, NJ, USA). Water (HPLC grade) was provided by Milli-Q plus purification system (Billerica, MA, USA) available at site. All chemicals are analytical grade and solvents are HPLC grade.

Ethical approval for the Animal experiments was acquired from the Animal Ethics Committee at King Saud University (No. KSU-SE-22-83). All animal experiments were completed following the standards of the experimental Animal Use and Care Guidelines of the National Institutes of Health and the Supervision of Animal Experiments Committee. Sprague-Dawley rats were acquired from experimental animal care center King Saud University (Riyadh, Saudi Arabia).

### 3.2. Chromatographic Conditions

6320 Ion Trap LC–MS^n^ (Agilent Technologies, Palo Alto, CA, USA) was used for the analysis of samples. Electrospray ionization was performed at room temperature in positive ion mode. The dry temperature was 350 °C, the nebulizer pressure was 60 psi, the capillary temperature was 325 °C, the dry gas was 10 L/min. The column used was an Eclipse plus C18 (4.6 × 150 mm, 3.5 micron) (Agilent Technologies, Palo Alto, CA, USA). LC separation was conducted using a mobile phase solvent A: water with 1% formic acid and solvent B: acetonitrile. The run began at 95% mobile phase A, then the percentage of mobile phase B was increased from 5 to 60% in 20 min and then increased to reach 65% at 25 min then kept at this percentage until minute 30. Gradient chromatography was performed with a mobile phase of a water/acetonitrile mixture with a flow rate of 0.4 mL/min and a total run time of 45 min. Injection volume of samples was 5 µL taken from vials that are placed in the autosampler. The indicated chromatographic parameters for investigation of FBB metabolites are summarized in Table 1.

### 3.3. In Silico Prediction of FBB Metabolites and Structural Alerts Using WhichP450™ Metabolism Module and DEREK NEXUS Module of StarDrop Software

StarDrop software was used to predict the main sites of metabolism and to predict site lability specified by the composite site lability (CSL). This software contains the WhichP450 module which predicts regioselectivity of metabolism by different isoforms. This module concludes the findings by a pie chart of the most likely CYP450 isoform that has a major role in FBB metabolism. DEREK module was used to identify structural alerts in FBB that can cause certain toxicities.

### 3.4. RLM Incubations

Protein concentration of the prepared RLMs was determined using Lowery method [35]. FBB was dissolved in dimethyl sulfoxide (DMSO). FBB (5 μM) was incubated with RLMs (1 mg/mL) in phosphate buffer (50 mM Na/K and 3.3 mM MgCl_2_) at pH 7.4. One mM NADPH was added to initiate the metabolic reaction. One mM of trapping agents KCN, GSH or methoxylamine were used in the experiments for capturing of iminium, iminopyridinone and aldehyde reactive intermediates, respectively. The reactions were performed in a thermostatic shaking water bath (37 °C for 60 min.). Two mL of ACN (ice cold) was added to stop the metabolic reaction by denaturation of enzymes protein. Centrifugation at 9000× *g* was done for 12 min at 4 °C to precipitate proteins. The clear supernatants were evaporated under a stream of nitrogen gas then reconstituted in mobile phase (50:50). Negative controls were prepared in the absence of RLMs or NADPH to confirm that FBB phase I metabolites were metabolically produced [28,36,37].

### 3.5. Characterization of FBB Reactive Intermediates

Extracted ion chromatograms (EIC) of various incubation mixtures were used to characterize FBB metabolites and reactive intermediates. Fragment ions were used to reconstruct the chemical structure of FBB metabolites and reactive intermediates.

## 4. Conclusions

This study involved in vitro (RLMs) metabolite characterization and bioactivation identification of FBB using LC-MS/MS. Ten in vitro phase I metabolites of FBB were identified (Figure 6). Different pathways of phase I metabolism of FBB were proposed including oxidation of primary alcohol to aldehyde, hydroxylation, N-oxidation and N-dealkylation. Four iminium reactive intermediates, five 6-iminopyridin-3(*6H*)-one reactive intermediates and six aldehyde reactive intermediates of FBB were identified. Piperazine moiety in FBB is predicted by DEREK software to cause toxicity and is proposed to bioactivated to iminium reactive intermediates captured by cyanide. Oxidation of the hydroxymethyl group on the pyridine ring is proposed to the cause the formation of reactive aldehyde intermediates captures by methoxylamine. N-dealkylation and hydroxylation of the pyridine ring is proposed to cause formation of 6-iminopyridin-3(*6H*)-one reactive intermediates captured by glutathione. FBB and several phase I metabolites are bioactivated to fifteen reactive intermediates which might be the cause of adverse effects.

## Figures and Tables

**Table 1:** Summary of selected liquid chromatography and mass spectrometry conditions.

Mobile phase.	Binary system of 0.1% Formic acid in H_2_O (A) and ACN (B)		ESI source	Positive ESI
				High purity N_2_ gas Drying gas at 10 L/min with pressure (60 psi)
	0.4 mL/min.			
	Elution time: 45 min.			
Agilent Zorbax eclipse plus C_18_ Column	Length	150 mm		ESI temperature: 350 °C
	ID	4.6 mm		Capillary voltage: 4000 V
	Particle size	3.5 μm	Modes	Mass scan, MS^2^ and MS^3^
	Temperature	22 ± 1 °C	Collision gas	High purity N_2_
Gradient elution system	Time in min.	% ACN	Analytes	FBB and its metabolites
	0	5	Mass parameters	Fragmentor voltage (FV): 145 V Amplitude: 1.25 V
	20	60		
	25	65		
	30	65		
	45	5		

## Data Availability

All data are available within the manuscript.

## Supplementary Materials

The following supporting information can be downloaded at: https://www.mdpi.com/article/10.3390/molecules28104225/s1, Figure S1: Product ion mass spectrum of M2 showing proposed fragmentation pattern; Figure S2: Product ion mass spectrum of M3 showing proposed fragmentation pattern; Figure S3: Product ion mass spectrum of M4 showing proposed fragmentation pattern; Figure S4: Product ion mass spectrum of M5 showing proposed fragmentation pattern; Figure S5: Product ion mass spectrum of M6 showing proposed fragmentation pattern; Figure S6: Product ion mass spectrum of M7 showing proposed fragmentation pattern; Figure S7: Product ion mass spectrum of M8 showing proposed fragmentation pattern; Figure S8: Product ion mass spectrum of M9 showing proposed fragmentation pattern; Figure S9: Product ion mass spectrum of M10 showing proposed fragmentation pattern; Figure S10: Product ion mass spectrum of M12/KCN cyanide adduct showing proposed fragmentation pattern; Figure S11: Product ion mass spectrum of M13/KCN cyanide adduct showing proposed fragmentation pattern; Figure S12: Product ion mass spectrum of M14/KCN cyanide adduct showing proposed fragmentation pattern; Figure S13: Product ion mass spectrum of M16/GSH adduct showing proposed fragmentation pattern; Figure S14: Product ion mass spectrum of M17/GSH adduct showing proposed fragmentation pattern; Figure S15: Product ion mass spectrum of M18/GSH adduct showing proposed fragmentation pattern; Figure S16: Product ion mass spectrum of M19/GSH adduct showing proposed fragmentation pattern; Figure S17: Product ion mass spectrum of M21/CH_3_ONH_2_ methoxyleamine adduct showing proposed fragmentation pattern; Figure S18: Product ion mass spectrum of M22/CH_3_ONH_2_ methoxyleamine adduct showing proposed fragmentation pattern; Figure S19: Product ion mass spectrum of M23/CH_3_ONH_2_ methoxyleamine adduct showing proposed fragmentation pattern; Figure S20: Product ion mass spectrum of M24/CH_3_ONH_2_ methoxyleamine adduct showing proposed fragmentation pattern; Figure S21: Product ion mass spectrum of M25/CH_3_ONH_2_ methoxyleamine adduct showing proposed fragmentation pattern; Table S1: In vitro phase 1 metabolites of FNB; Table S2: Summary of proposed cyanide adducts of FNB; Table S3: Summary of proposed GSH adducts of FNB; Table S4: Summary of proposed methoxylamine adducts of FNB.

## Abbreviations

FBB, Fenebrutinib; ACN, acetonitrile; conc. RLM, rat liver microsomes; KCN, potassium cyanide; BTK, Bruton tyrosine kinase; DEREK, StarDrop WhichP450™ module, reactive intermediates, phase I metabolites, GSH, glutathione; KCN, potassium cyanide.
