# Supplementary material for: Investigation of Fenebrutinib Metabolism and Bioactivation Using MS3 Methodology in Ion Trap LC/MS
Source: Molecules. 2023 May 22;28(10):4225. doi: 10.3390/molecules28104225 (PMC10220799; doi:10.3390/molecules28104225)
Supplement: Supplementary file 1 [file molecules-28-04225-s001.zip › molecules-2350158-supplementary.pdf]

Investigation of Fenebrutinib Metabolism and Bioactivation Using MS3 Methodology in Ion Trap LC/MS

Supplementary data

Aishah M. Alsibaee, Haya I. Aljohar, Mohamed W. Attwa *, Ali S. Abdelhameed
and Adnan A. Kadi

- **Identification of M2.**

M2 (*m/z* 663) is proposed to be generated by oxidation of primary alcohol to aldehyde of FNB. This metabolite's peak elutes at 21.2 minute in fragment ion chromatogram. Dissociation of M2 ion at inside the collision cell generates five fragment ions at *m/z* 645, *m/z* 635, *m/z* 604, *m/z* 588 and m/*z* 414 (Figure S1).

A

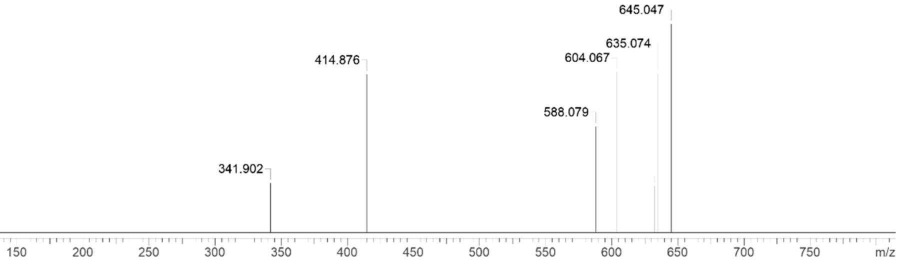

B

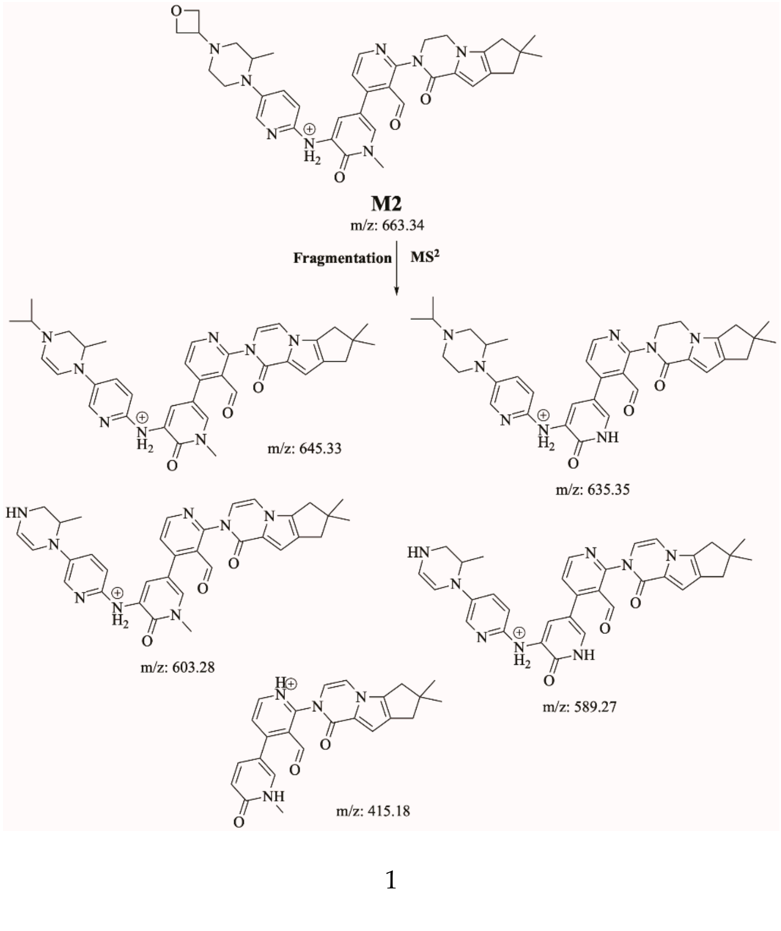

Figure S1. A) Product ion mass spectrum of M2, B) Proposed interpretation of fragmentation of M2.

- **Identification of M3.**

M3 (*m/z* 609) is proposed to be generated by N-dealkylation of FNB. This metabolite's peak elutes at 22.63 minute in fragment ion chromatogram. Dissociation of M3 ion inside the collision cell generates four fragment ions at *m/z* 591, *m/z* 551, *m/z* 471*, m/z* 276 and *m/z* 366 (Figure S2).

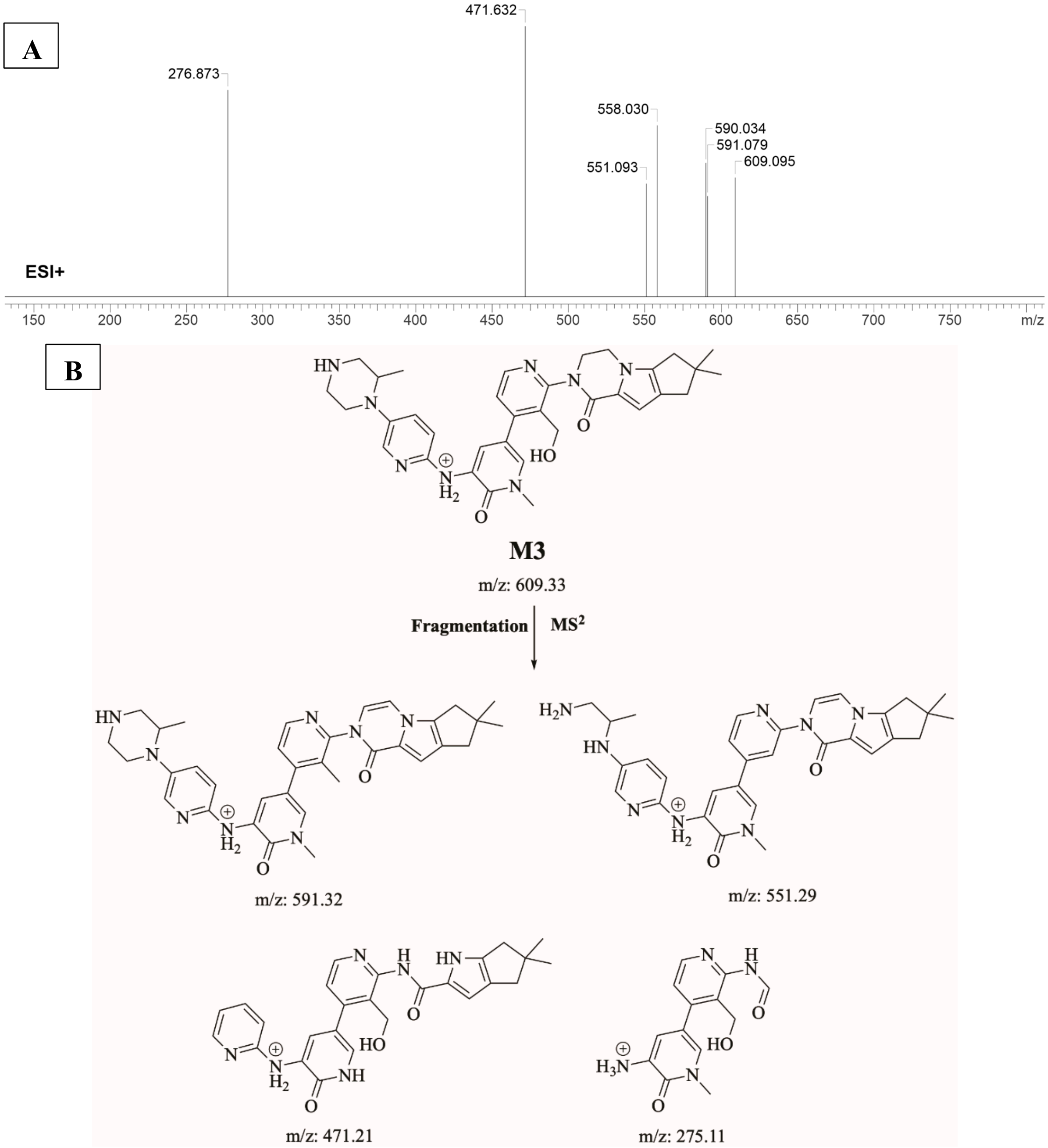

Figure S2. A) Product ion mass spectrum of M3, B) Proposed interpretation of fragmentation of M3.

- **Identification of M4.**

M4 (m/z 651) is proposed to be generated by N-demethylation of tertiary carboxamide of FNB. This metabolite's peak elutes at 20.04 minute in fragment ion chromatogram. Dissociation of M4 ion inside the collision cell generates six fragment ions at *m/z* 619, *m/z* 591, *m/z* 509 and *m/z* 392 (Figure S3).

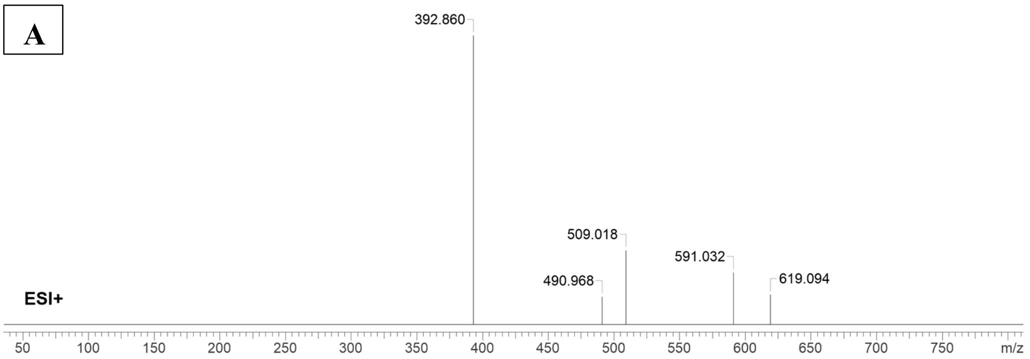

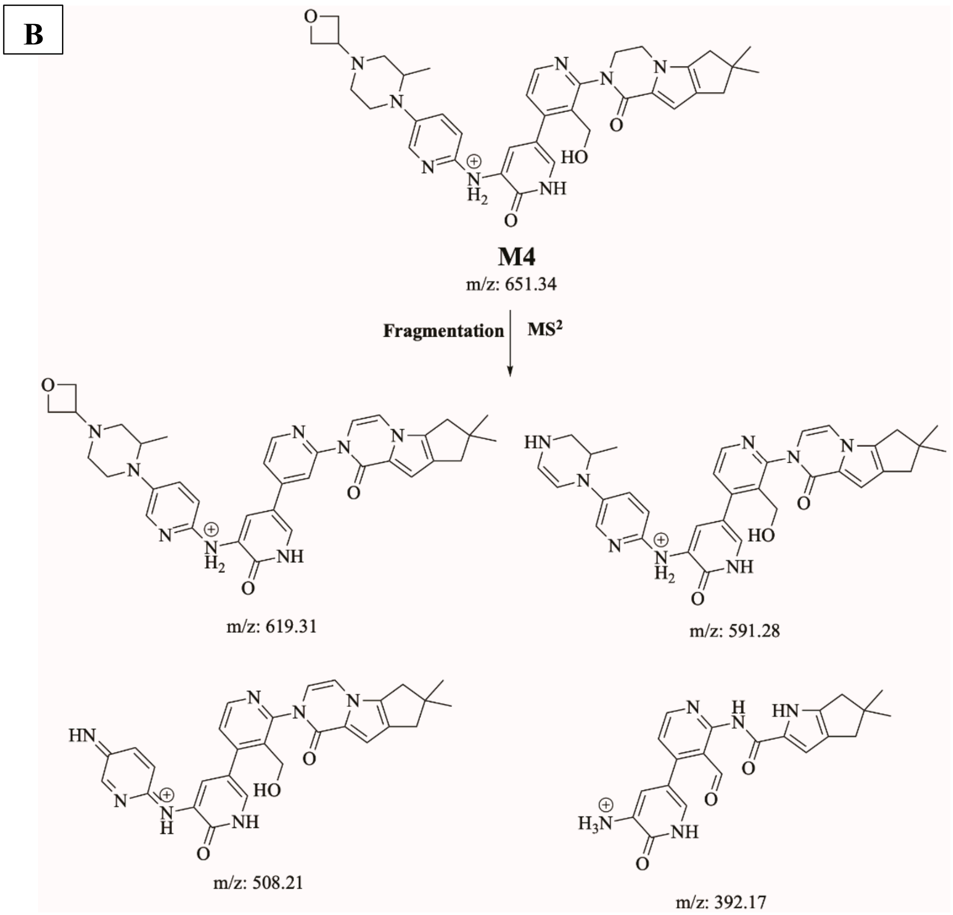

Figure S3. A) Product ion mass spectrum of M4, B) Proposed interpretation of fragmentation of M4.

- **Identification of M5.**

M5 (m/z 697) is proposed to be generated by hydroxylation and N-oxidation of FNB. This metabolite's peak elutes at 24 minutes in fragment ion chromatogram. Dissociation of M5 ion inside the collision cell generates five fragment ions at *m/z* 679, *m/z* 646*, m/z 628, m/z* 607 and *m/z* 479 (Figure S4).

A

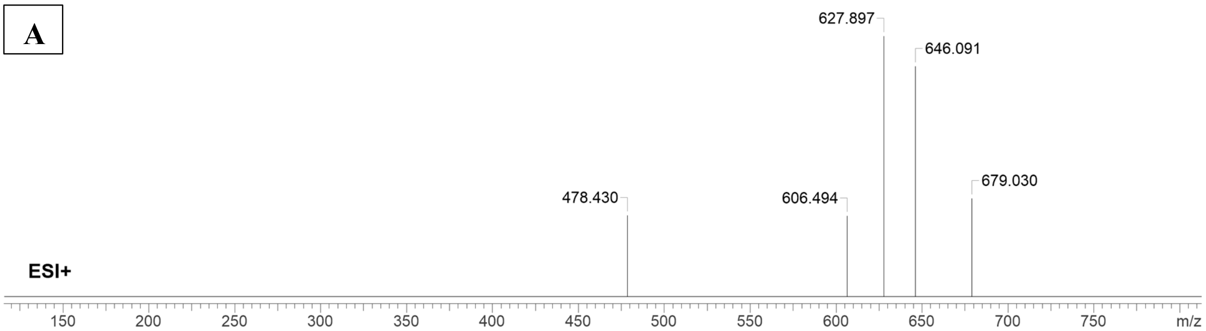

B

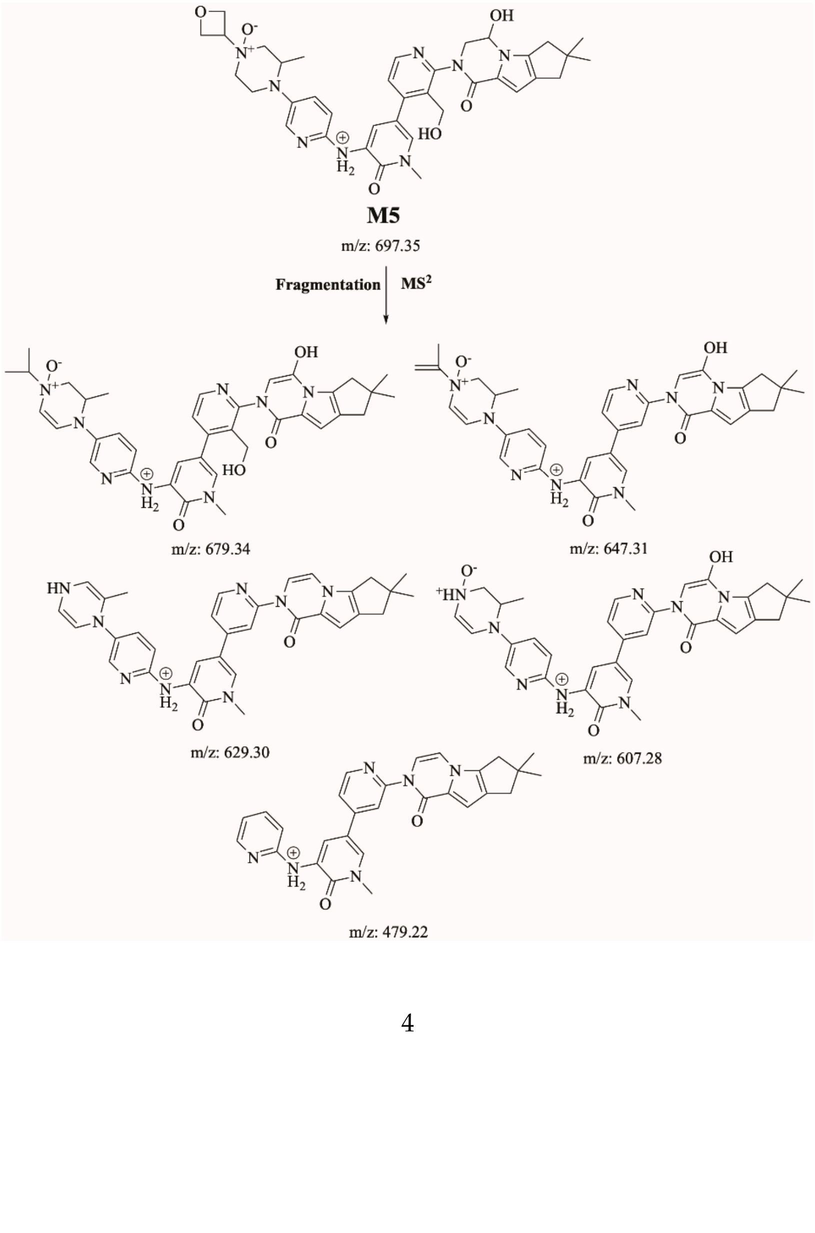

Figure S4. A) Product ion mass spectrum of M5, B) Proposed interpretation of fragmentation of M5.

- **Identification of M6.**

M6 (m/z 679) is proposed to be generated by opening of piperazine ring and oxidation of FNB. This metabolite's peak elutes at 24 minutes in fragment ion chromatogram. Dissociation of M6 ion inside the collision cell generates six fragment ions at *m/z* 661, *m/z 633, m/z 593, m/z* 430, *m/z* 364 and *m/z* 351 (Figure S5).

A

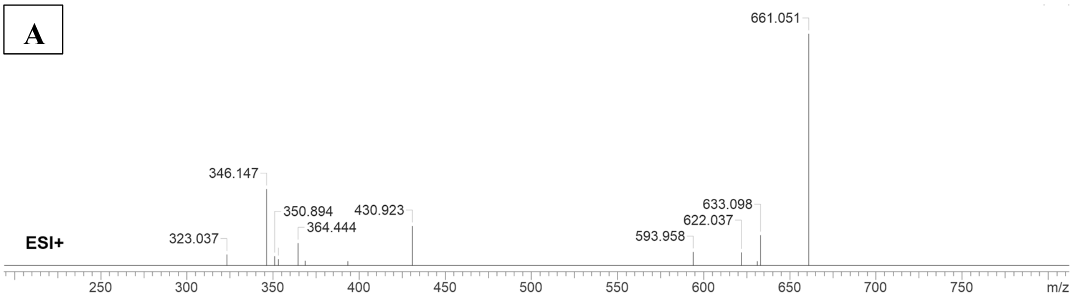

B

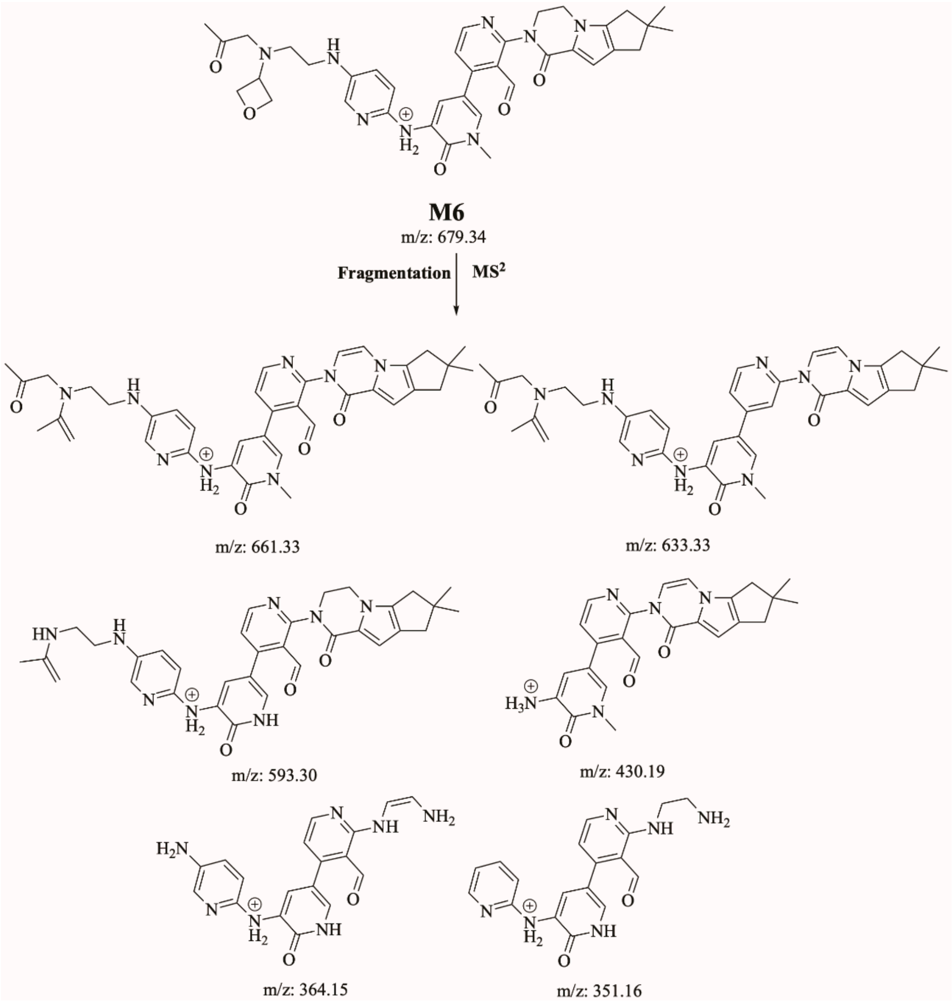

Figure S5. A) Product ion mass spectrum of M6, B) Proposed interpretation of fragmentation of M6.

- **Identification of M7.**

M7 (m/z 679) is proposed to be generated by oxidation of primary alcohol to aldehyde and hydroxylation of FNB. This metabolite's peak elutes at 18 minutes in fragment ion chromatogram. Dissociation of M7 ion inside the collision cell generates five fragment ions at *m/z 661, m/z 643 m/z 563, m/z* 436 and *m/z* 453 (Figure S6).

A

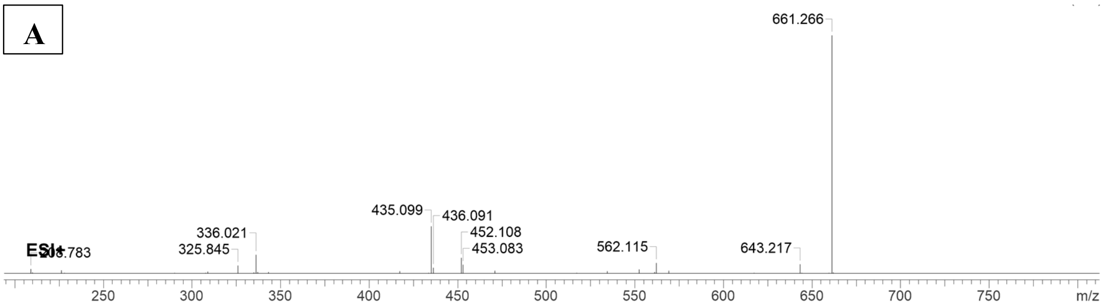

B

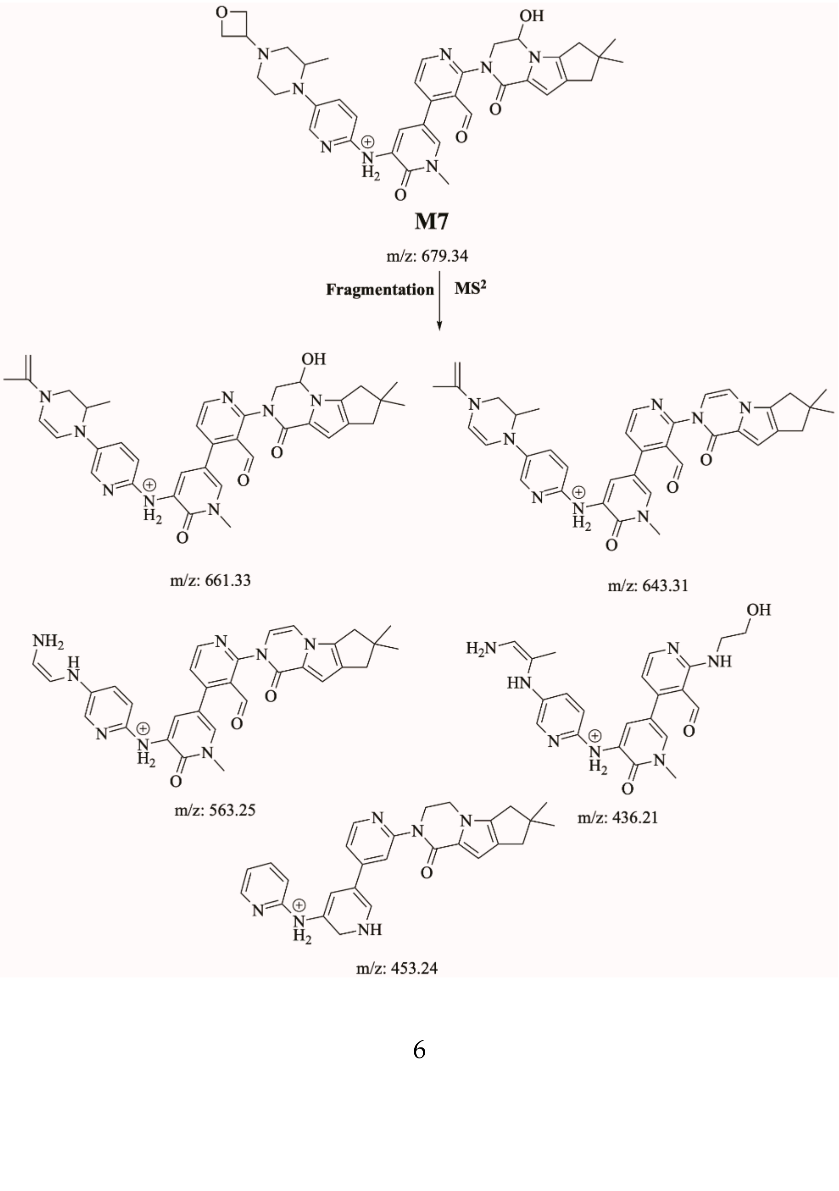

Figure S6. A) Product ion mass spectrum of M7, B) Proposed interpretation of fragmentation of M7.

- **Identification of M8.**

M8 (m/z 681) is proposed to be generated by N-oxidation of FNB. This metabolite's peak elutes at 15.9 minute in fragment ion chromatogram. Dissociation of M8 ion at *m/z* 681 inside the collision cell produces one fragment at *m/z* 663 (loss of water molecule). Further investigation using MS^3 analysis of fragment *m/z* 476 yielded four characteristic and qualitative fragment ions at *m/z 633 m/z 619, m/z* 484 and *m/z* 415 (Figure S7).

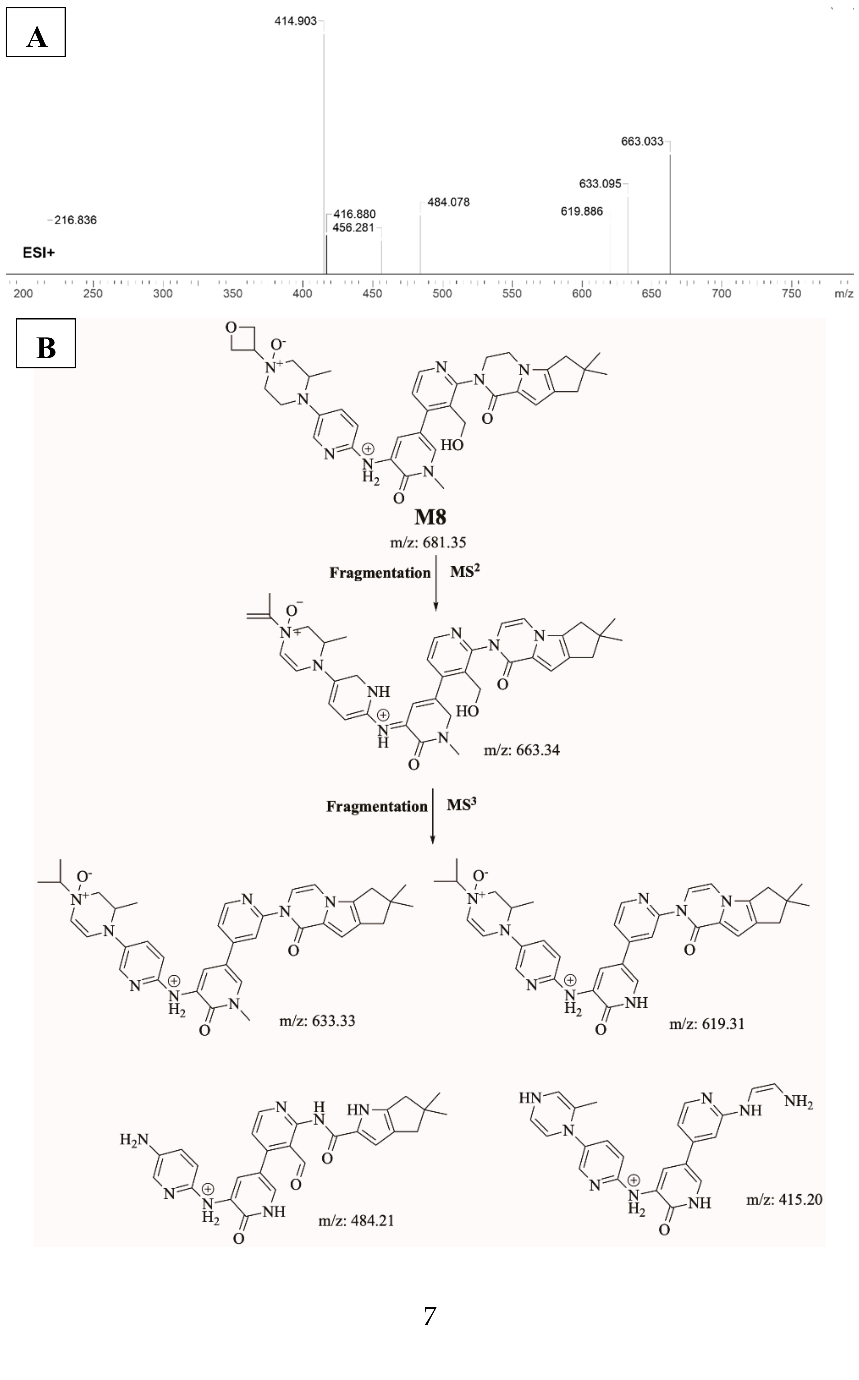

Figure S7. A) Product ion mass spectrum of M8, B) Proposed interpretation of fragmentation of M8.

- **Identification of M9.**

M9 (m/z 681) is proposed to be generated by opening and oxidation of piperazine ring of FNB. This metabolite's peak elutes at 16.93 minute in fragment ion chromatogram. Dissociation of M9 ion at *m/z* 681 inside the collision cell produces one fragment at *m/z* 663 (loss of water molecule). Further investigation using MS^3 analysis of fragment *m/z* 663 yielded four characteristic and qualitative fragment ions at *m/z 633 m/z 619, m/z* 484 and *m/z* 415 (Figure S8).

A

B

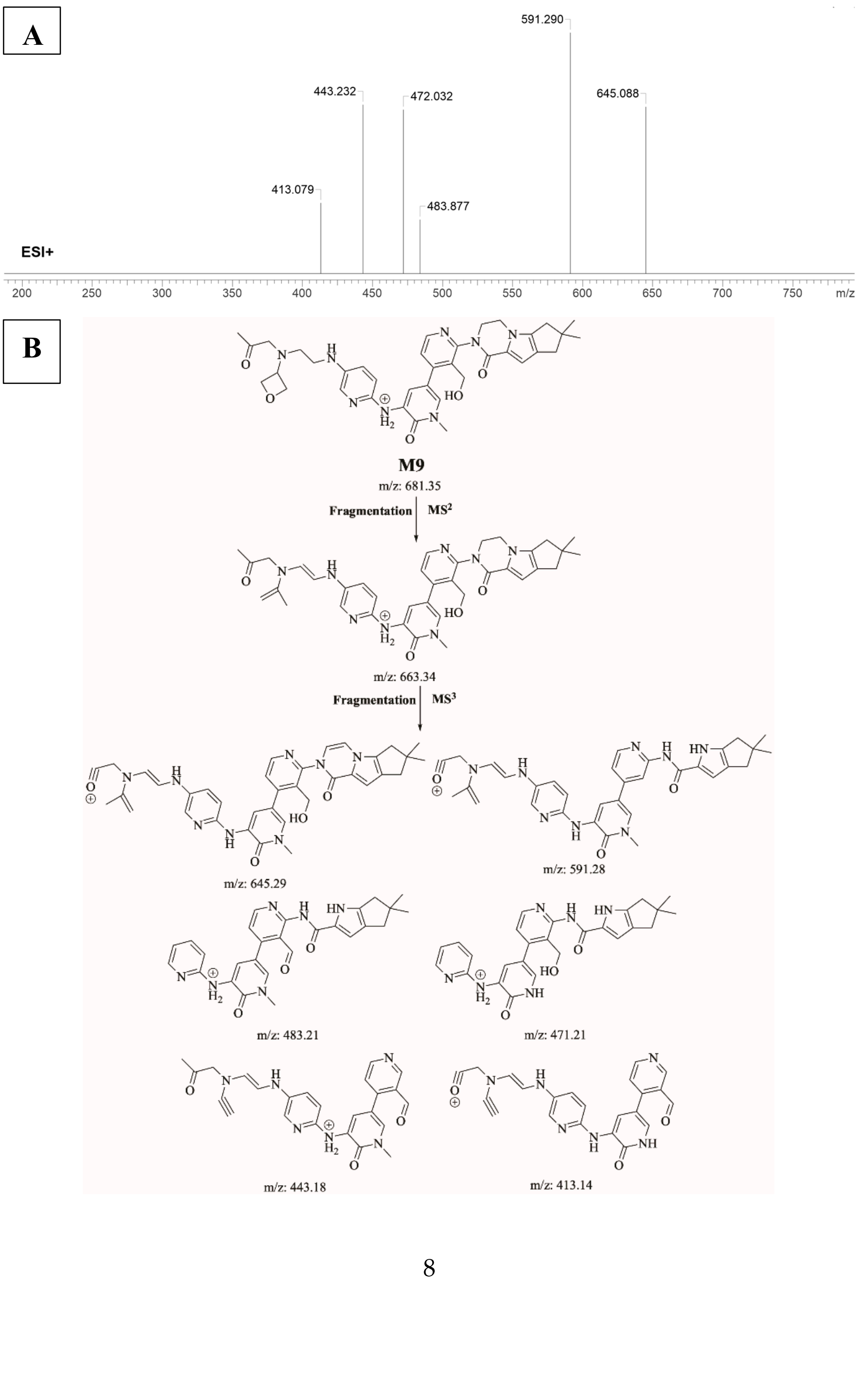

Figure S8. A) Product ion mass spectrum of M9, B) Proposed interpretation of fragmentation of M9.

- **Identification of M10.**

M10 (m/z 681) is proposed to be generated by hydroxylation of FNB. This metabolite's peak elutes at 18.8 minute in fragment ion chromatogram. Dissociation of M10 ion at *m/z* 681 inside the collision cell produces one fragment at *m/z* 663 (loss of water molecule). Further investigation using MS^3 analysis of fragment *m/z* 663 yielded four characteristic and qualitative fragment ions at *m/z 645 m/z 535, m/z 455m/z 436* and *m/z* 336 (Figure S9).

A

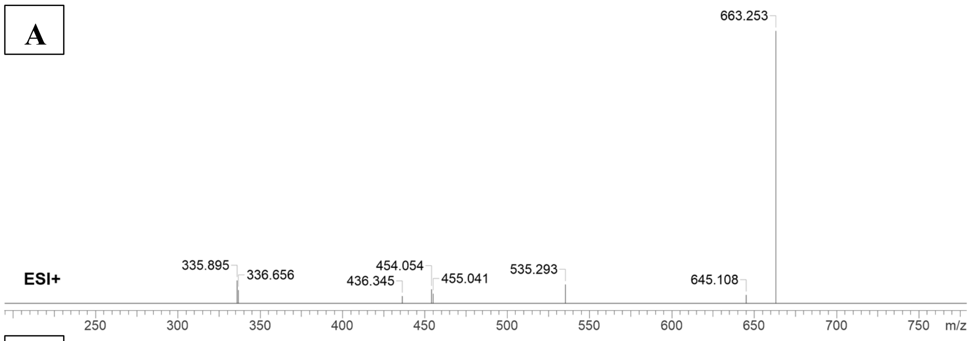

B

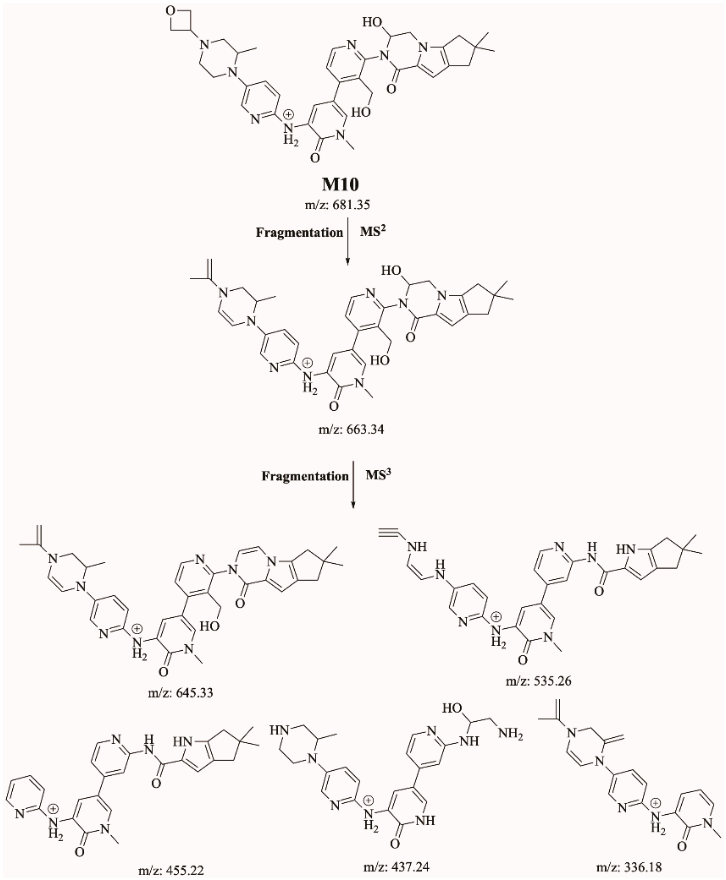

Figure S9. A) Product ion mass spectrum of M10, B) Proposed interpretation of fragmentation of M10.

Table S1. *In vitro* phase 1 metabolites of FNB.

	m/z	Main fragment (MS^3)	Fragments	Retention time	Proposed metabolic reaction
M1	494	476	464,380,322.	18.39	Hydroxylation and N-dealkylation.
M2	663	NA	645, 635, 604, 588, 414.	21.2	Oxidation of primary alcohol to aldehyde.
M3	609	NA	591, 551, 471, 276.	22.63	N-dealkylation.
M4	651	NA	591, 531, 509, 392.	20.04	N-demethylation.
M5	697	NA	679, 646, 628, 607, 478.	24	N-oxidation and hydroxylation.
M6	679a	NA	661, 633, 622, 593, 430, 364, 351.	24	Oxidation and opening of piperazine ring and oxidation of primary alcohol to aldehyde.
M7	679b	NA	661, 643, 562, 453, 436.	18	Oxidation of primary alcohol to aldehyde and hydroxylation.
M8	681a	663	663, 633, 619, 484, 415.	15.9	N-oxidation.
M9	681b	663	645, 591, 483, 471, 443, 413.	16.93	Oxidation and opening of piperazine ring.
M10	681c	663	663, 645, 535, 455, 436, 336, 335.	18.8	Hydroxylation.

- **Identification of M12/KCN cyanide adduct.**

M12/KCN is proposed to form by the addition of cyanide group to M3 of FNB and oxidation of primary alcohol to aldehyde. M12/KCN (*m/z* 632) peak appeared at 18.95 minute in fragment ion chromatogram. Dissociation of M12/KCN ion inside the collision cell produces five fragment ions at m/z 565, m/z 563, m/z 432, m/z 369 and m/z 363 (Figure S10).

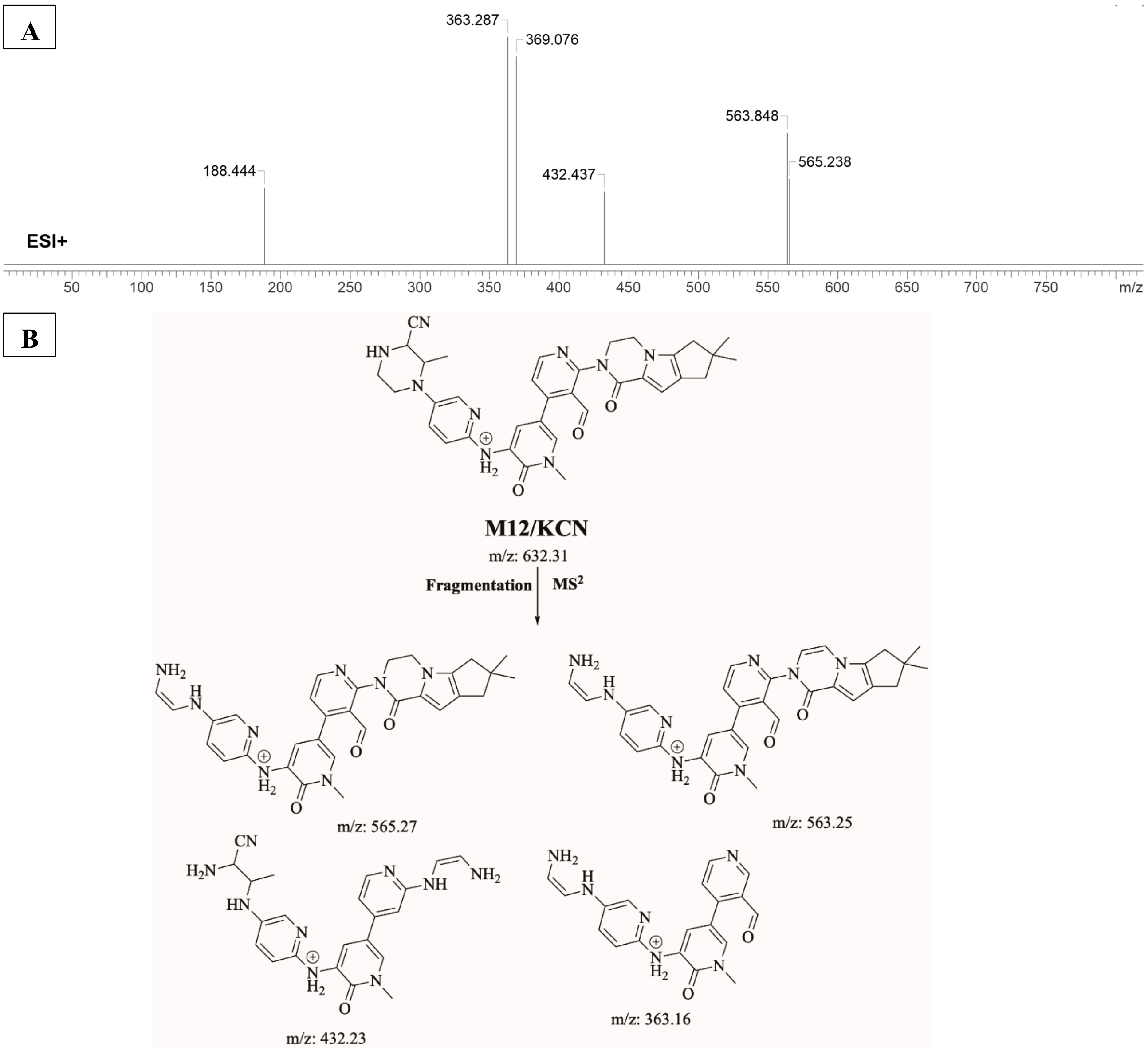

Figure S10. A) Product ion mass spectrum of M12/KCN cyanide adduct, B) Proposed interpretation of fragmentation of M12/KCN.

- **Identification of M13/KCN cyanide adduct.**

M13/KCN is proposed to form after double N-dealkylation and hydroxylation of FNB. M13/KCN (*m/z* 463) peak appeared at 21.12 minute in fragment ion chromatogram. Dissociation of M13/KCN ion inside the collision cell produces four fragment ions at m/z 445, m/z 416, m/z 402 and m/z 323 (Figure S11).

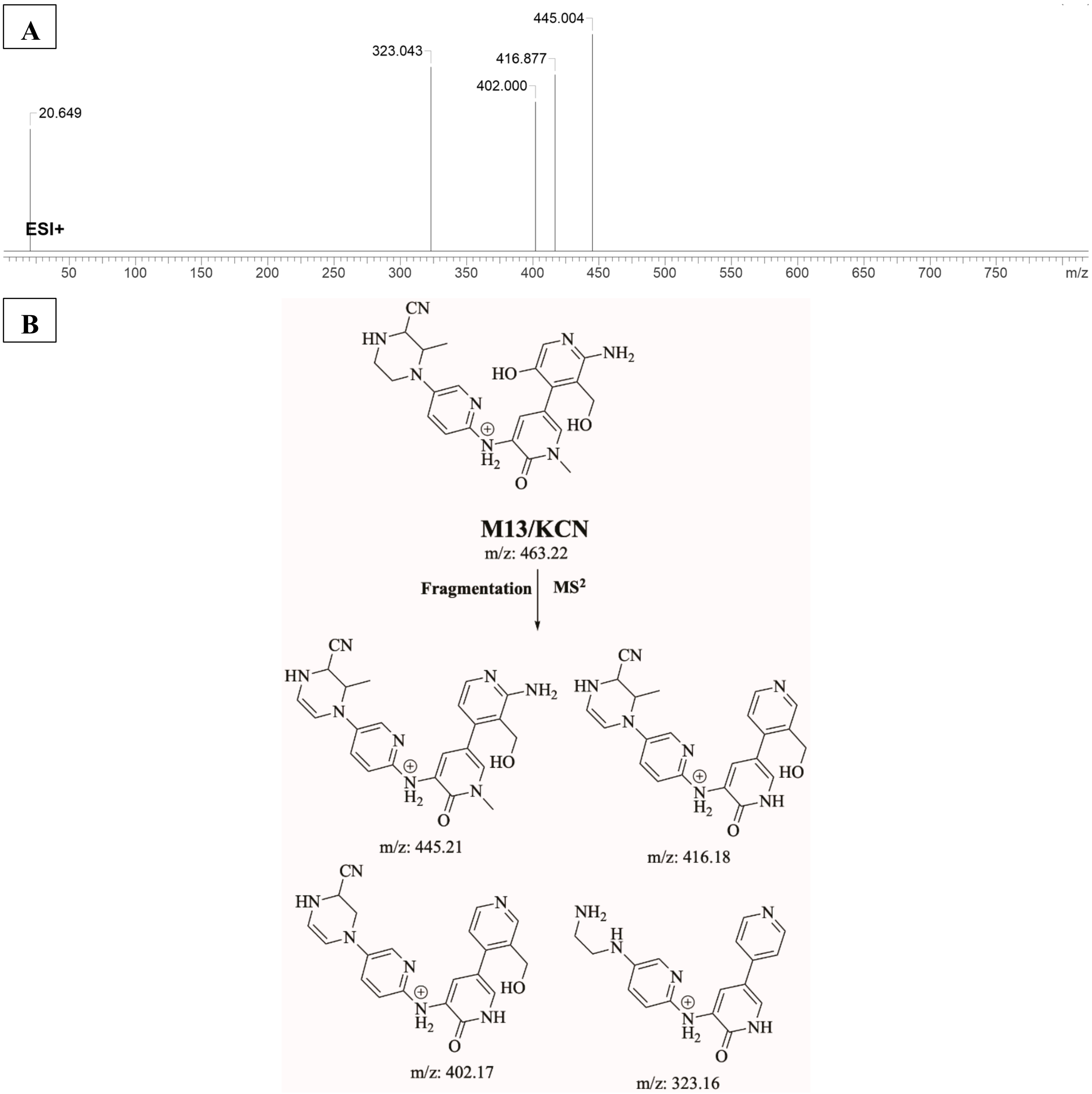

Figure S11. A) Product ion mass spectrum of M13/KCN cyanide adduct, B) Proposed interpretation of fragmentation of M13/KCN.

- **Identification of M14/KCN cyanide adduct.**

M14/KCN is proposed to form by the addition of cyanide group after double N-dealkylation of FNB. M14/KCN (*m/z* 447) peak appeared at 21.82 minutes in fragment ion chromatogram. Dissociation of M14/KCN ion inside the collision cell produces four fragment ions at m/z 420, m/z 352 m/z 325 and m/z 300 (Figure S12).

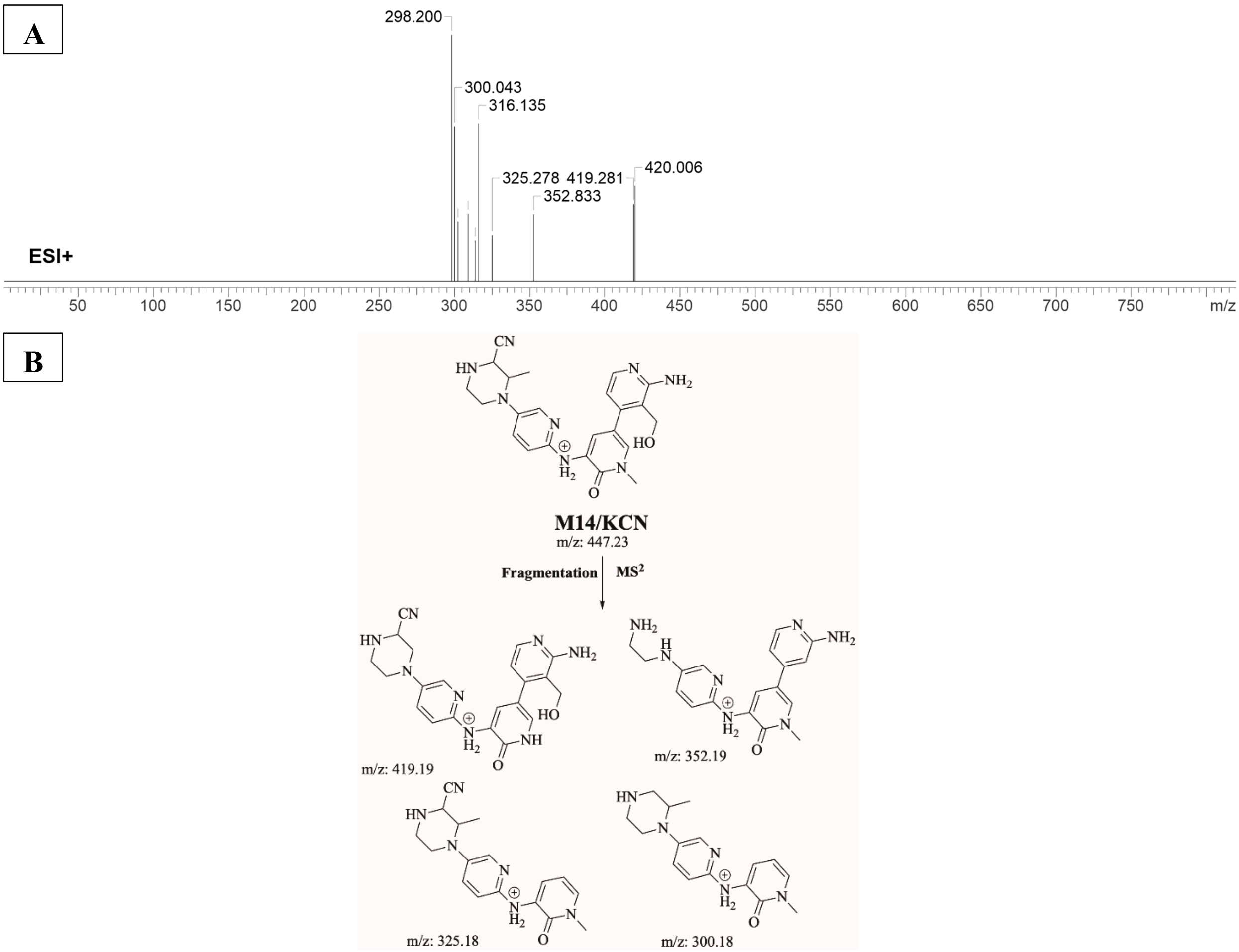

Figure S12. A) Product ion mass spectrum of M14/KCN cyanide adduct, B) Proposed interpretation of fragmentation of M14/KCN.

Table S2. Summary of proposed cyanide adducts of FNB.

	m/z	Fragments	Retention time	Proposed metabolic reaction
M11/KCN	620	525, 457, 347, 267.	16.94	N-dealkylation and N-demethylation.
M12/KCN	632	565, 563, 432, 369, 363, 188	18.95	N-dealkylation.
M13/KCN	463	445, 416, 402, 323.	21.12	Double N-dealkylation and hydroxylation.
M14/KCN	447	420, 419, 352, 316, 325, 300.	21.82	Double N-dealkylation.

- **Identification of M16/GSH adduct**

M16/GSH is proposed to form by the addition of GSH after double N-dealkylation and hydroxylation of FNB. M16/GSH (*m/z* 741) peak appeared at 18.43 minute in fragment ion chromatogram. Dissociation of FNB741 ion at *m/z* 741 inside the collision cell produces one fragment at *m/z* 723 (loss of water molecule). Further investigation using MS^3 analysis of fragment *m/z* 723 yielded four characteristic and qualitative fragment ions at *m/z 706, m/z 597, m/z 427* and *m/z* 335 (Figure S13).

A

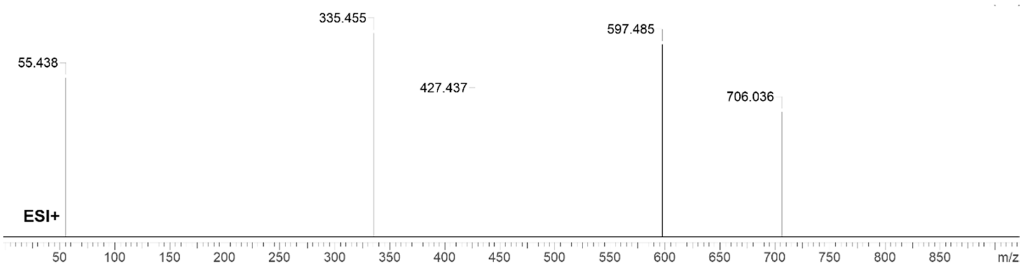

B

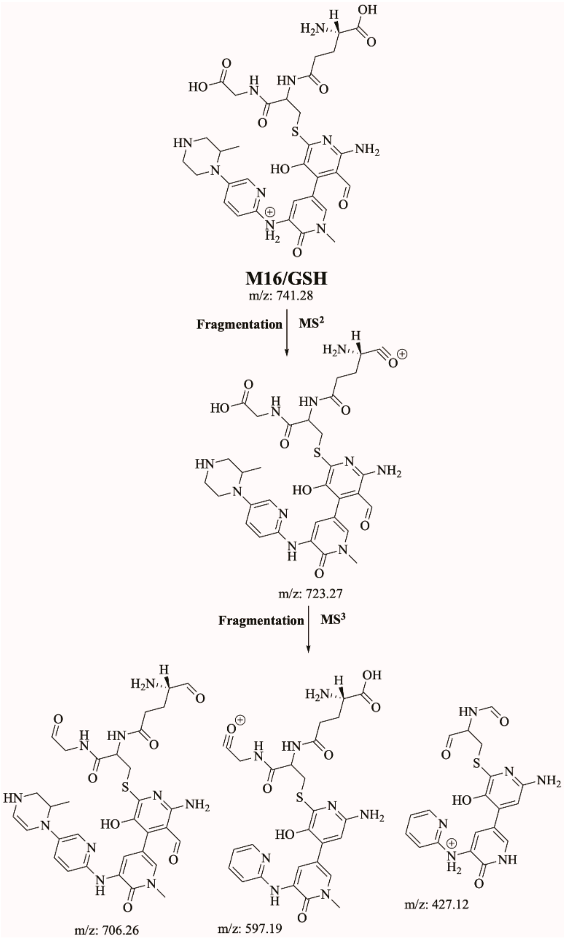

Figure S13. A) Product ion mass spectrum of M16/GSH adduct, B) Proposed interpretation of fragmentation of M16/GSH.

- **Identification of M17/GSH adduct**

M17/GSH is proposed to form by the addition of GSH after double N-dealkylation and hydroxylation of FNB. M17/GSH (*m/z* 743) peak appeared at 21.37 minute in fragment ion chromatogram. Dissociation of M17/GSH ion at *m/z* 743 inside the collision cell produces one fragment at *m/z* 684. Further investigation using MS^3 analysis of fragment *m/z* 684 yielded three characteristic and qualitative fragment ions at *m/z 588, m/z 565* and *m/z* 409 (Figure S14).

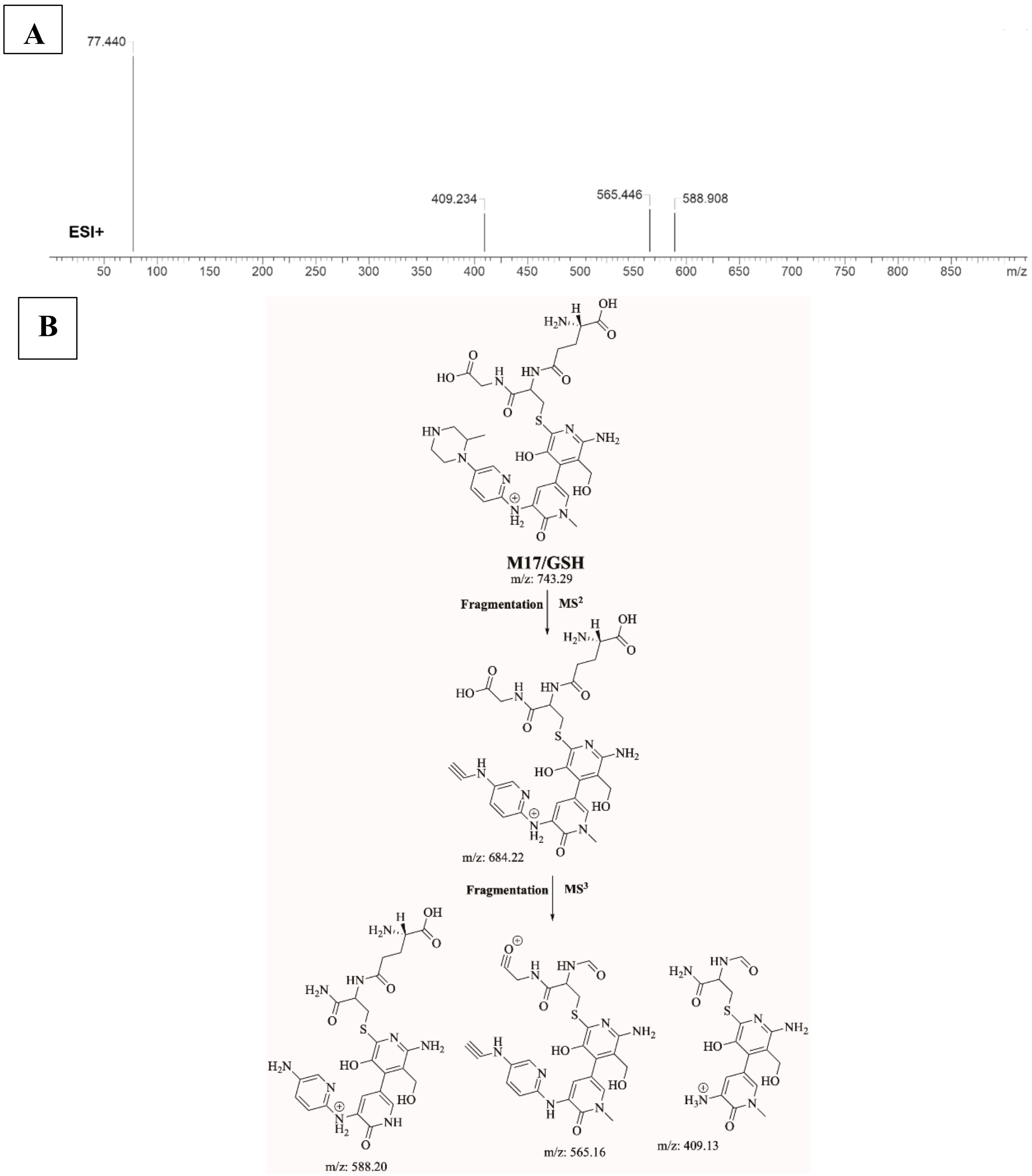

Figure S14. A) Product ion mass spectrum of M17/GSH adduct, B) Proposed interpretation of fragmentation of M17/GSH.

- **Identification of M18/GSH adduct**

M18/GSH is proposed to form by the addition of after N-dealkylation, oxidation of primary alcohol to aldehyde, hydroxylation and piperazine ring opening of FNB. M18/GSH (*m/z* 813) peak appeared at 17.47 minute in fragment ion chromatogram. Dissociation of M18/GSH ion at *m/z* 813 inside the collision cell produces one fragment at *m/z* 795 (loss of water molecule). Further investigation using MS^3 analysis of fragment *m/z* 795 yielded five characteristic and qualitative fragment ions at *m/z 673, m/z 655, m/z 399, m/z 266* and *m/z 251* (Figure S15).

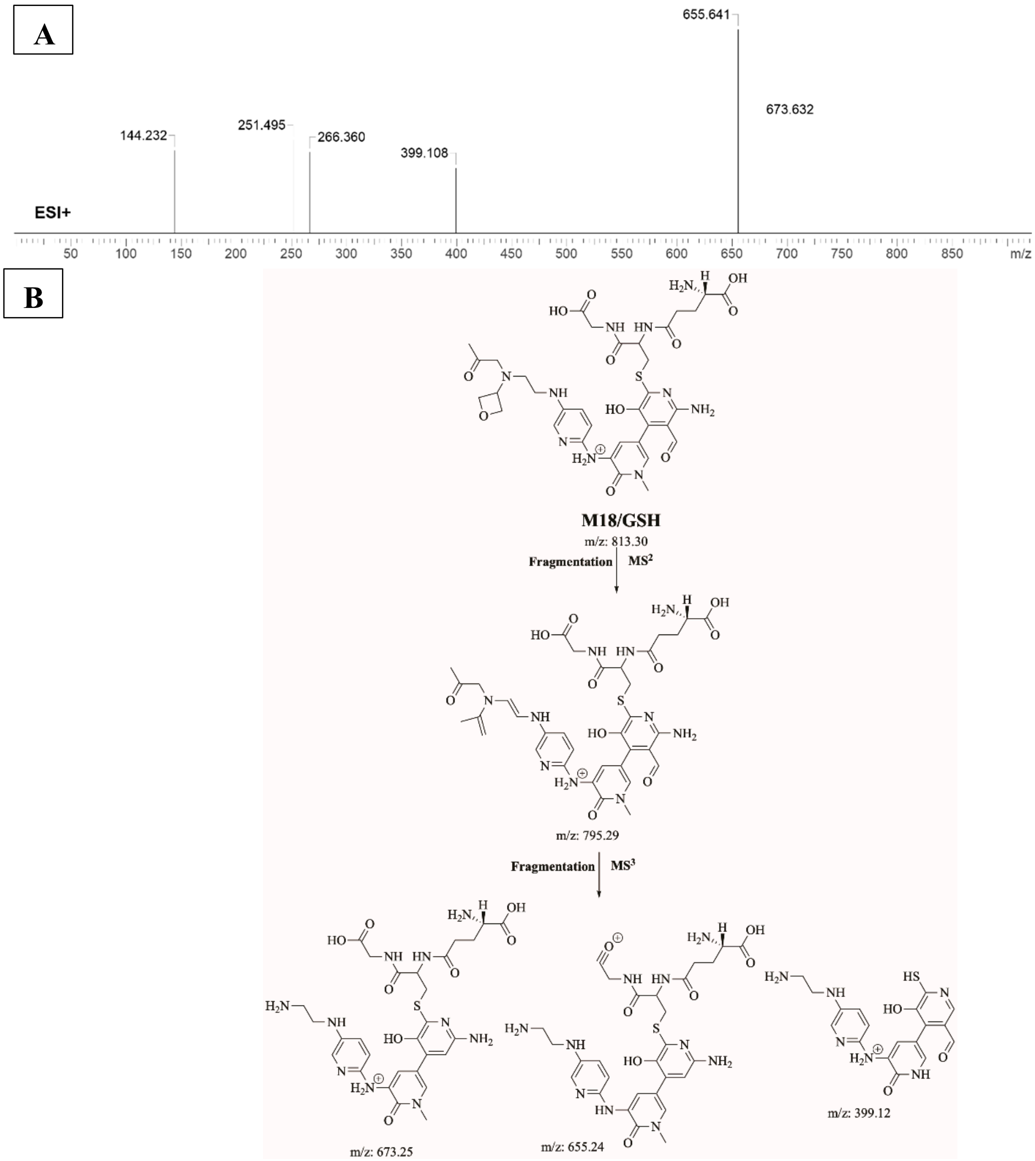

Figure S15. A) Product ion mass spectrum of M18/GSH adduct, B) Proposed interpretation of fragmentation of M18/GSH.

- **Identification of M19/GSH adduct**

M19/GSH is proposed to form by the addition of GSH after N-dealkylation, N-oxidation and hydroxylation of FNB. M19/GSH (*m/z* 815) peak appeared at 17.23 minute in fragment ion chromatogram. Dissociation of M19/GSH ion at *m/z* 815 inside the collision cell produces one fragment at *m/z* 729 (loss of trimethylene oxide ring). Further investigation using MS^3 analysis of fragment *m/z* 729 yielded five characteristic and qualitative fragment ions at *m/z 693, m/z 682, m/z 543, m/z 522* and *m/z 424* (Figure S16).

A

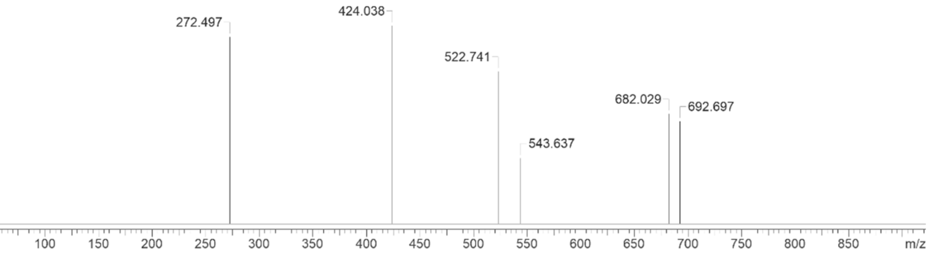

B

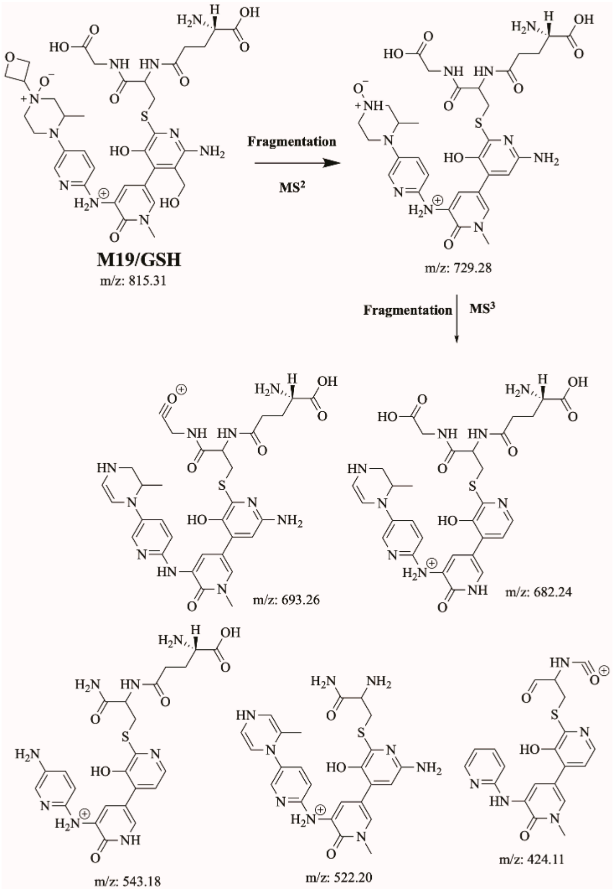

Figure S16. A) Product ion mass spectrum of M19/GSH adduct, B) Proposed interpretation of fragmentation of M19/GSH.

Table S3. Summary of proposed GSH adducts of FNB.

	m/z	Mian fragment (MS^3)	Fragments	Retention time	Proposed metabolic reaction
M15/GSH	799	739	725, 699, 609, 454, 252, 203.	17.28	N-dealkylation, and hydroxylation.
M16/GSH	741	723	706, 597, 427, 335.	18.43	Double n-dealkylation, hydroxylation and oxidation of primary alcohol to aldehyde.
M17/GSH	743	684	588, 565, 409.	21.37	Double n-dealkylation and hydroxylation.
M18/GSH	813	795	673, 655, 399, 267, 252.	17.47	Oxidation, opening of piperazine ring, oxidation of primary alcohol to aldehyde and hydroxylation.
M19/GSH	815	729	692, 682, 543, 522, 424, 272.	17.23	N-dealkylation, hydroxylation and N-oxidation.

- **Identification of M21/CH_3ONH_2 methoxylamine adduct**

M21/CH_3ONH_2 is proposed to form by the addition of methoxylamine to the aldehyde reactive metabolite of M1 of FNB. M21/CH_3ONH_2 (*m/z* 521) peak appeared at 17.01 minute in fragment ion chromatogram. Dissociation of M21/CH_3ONH_2 ion at *m/z* 521 inside the collision cell produces one fragment at *m/z* 475. Further investigation using MS^3 analysis of fragment *m/z* 475 yielded two characteristic and qualitative fragment ions at *m/z* 456 and *m/z* 283 (Figure S17).

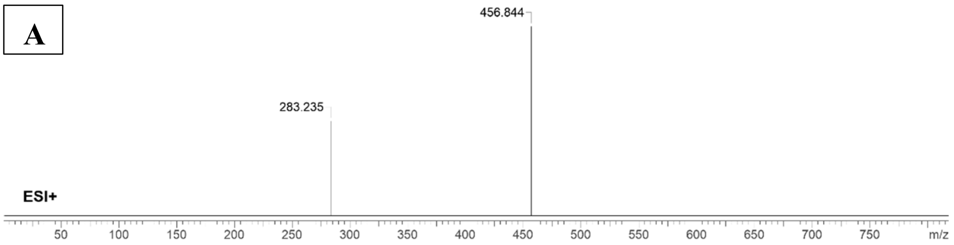

B

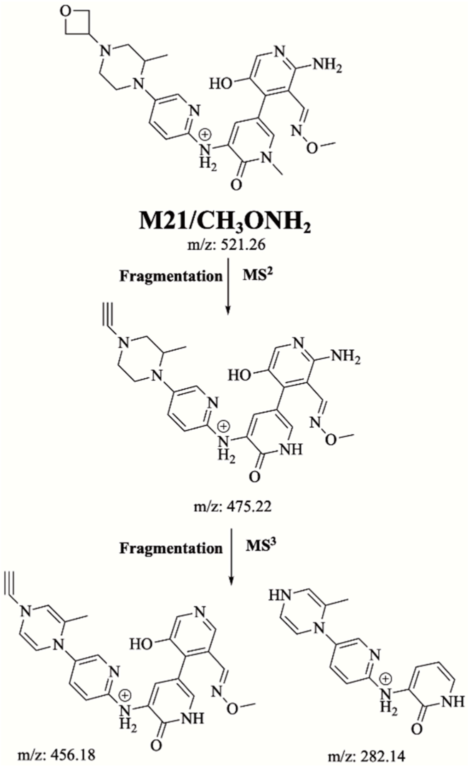

Figure S17. A) Product ion mass spectrum of M21/CH_3ONH_2 methoxyleamine adduct, B) Proposed interpretation of fragmentation of M21/CH_3ONH_2.

- **Identification of M22/CH_3ONH_2 methoxylamine adduct**

M22/CH_3ONH_2 is proposed to form by the addition of methoxylamine to the aldehyde reactive metabolite of M3 of FNB. M22/CH_3ONH_2 (*m/z* 521) peak appeared at 17.01 minute in fragment ion chromatogram. Dissociation of M22/CH_3ONH_2 ion at *m/z* 521 inside the collision cell produces one fragment at *m/z* 475. Further investigation using MS^3 analysis of fragment *m/z* 475 yielded two characteristic and qualitative fragment ions at *m/z* 456 and *m/z* 283 (Figure S18).

A

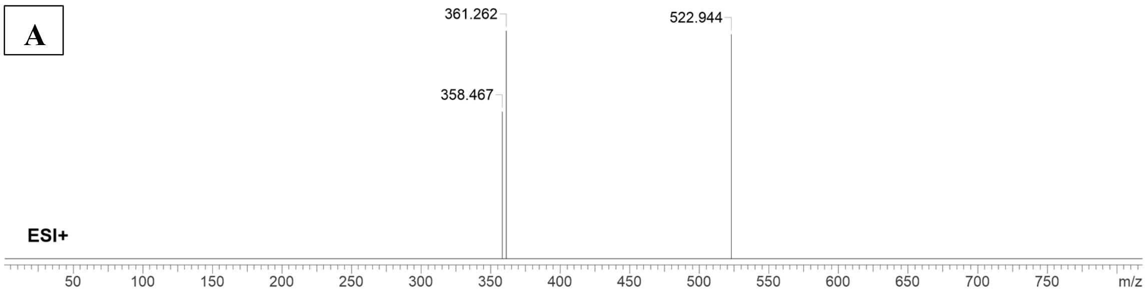

B

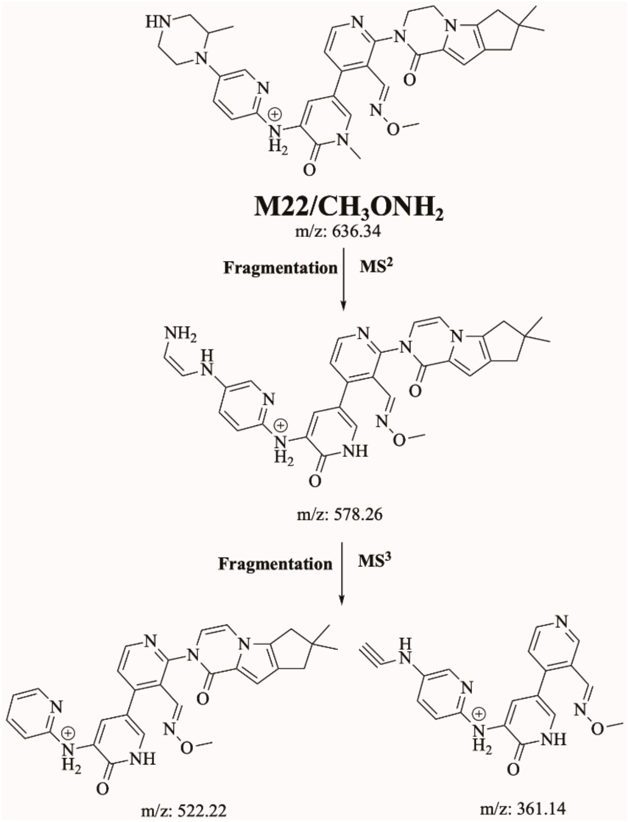

Figure S18. A) Product ion mass spectrum of M22/CH_3ONH_2 methoxylamine adduct, B) Proposed interpretation of fragmentation of M22/CH_3ONH_2.

- **Identification of M23/CH_3ONH_2 methoxylamine adduct**

M23/CH_3ONH_2 is proposed to form by the addition of methoxylamine to the aldehyde reactive metabolite of M6 of FNB. M23/CH_3ONH_2 (*m/z* 708) peak appeared at 22.99 minute in fragment ion chromatogram. Dissociation of M23/CH_3ONH_2 ion at *m/z* 708 inside the collision cell produces one fragment at *m/z* 638. Further investigation using MS^3 analysis of fragment *m/z* 638 yielded five characteristic and qualitative fragment ions at *m/z* 616, *m/z* 606, *m/z* 565, *m/z* 335 and *m/z* 240 (Figure S19).

A

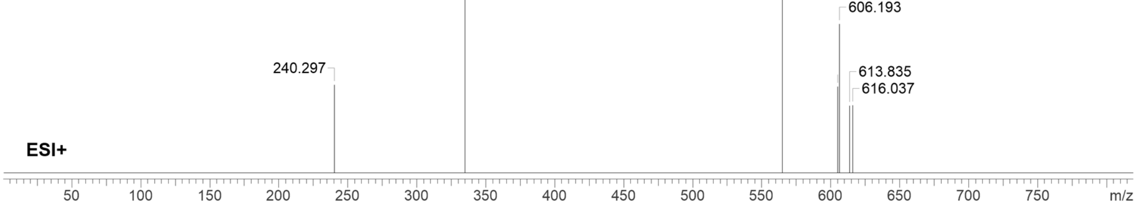

B

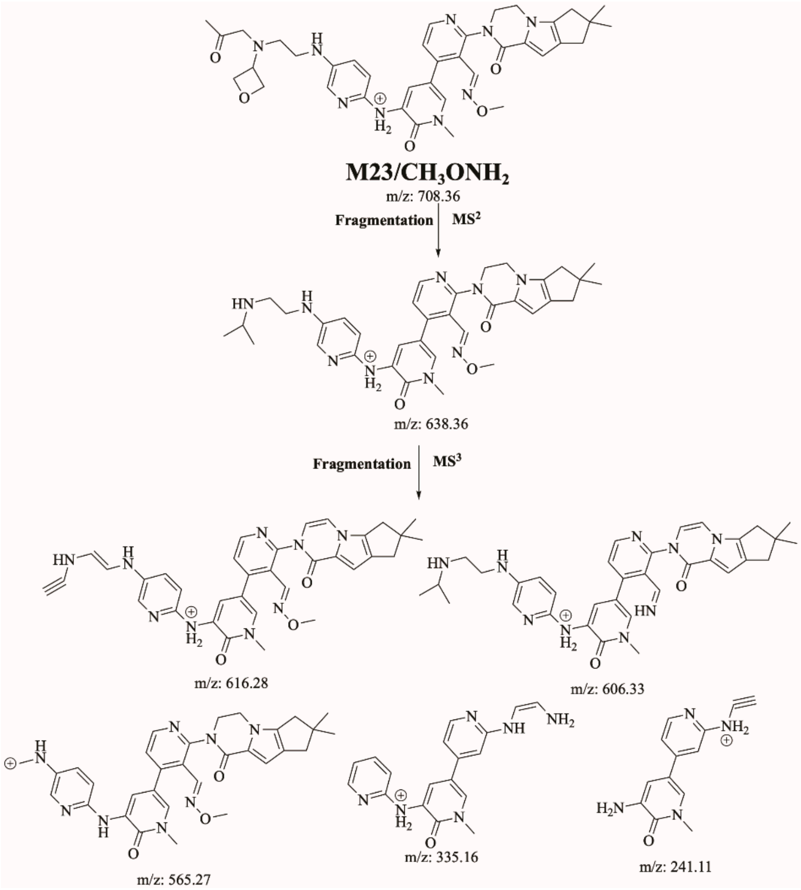

Figure S19. A) Product ion mass spectrum of M23/CH_3ONH_2 methoxylamine adduct, B) Proposed interpretation of fragmentation of M23/CH_3ONH_2.

- **Identification of M24/CH_3ONH_2 methoxylamine adduct**

M24/CH_3ONH_2 is proposed to form by the addition of methoxylamine to the aldehyde reactive metabolite of M5 of FNB. M24/CH_3ONH_2 (*m/z* 724) peak appeared at 17.6 minute in fragment ion chromatogram. Dissociation of M24/CH_3ONH_2 ion at *m/z* 724 inside the collision cell produces one fragment at *m/z* 706. Further investigation using MS^3 analysis of fragment *m/z* 706 yielded three characteristic and qualitative fragment ions at *m/z* 665, *m/z* 638 and *m/z* 395 (Figure S20).

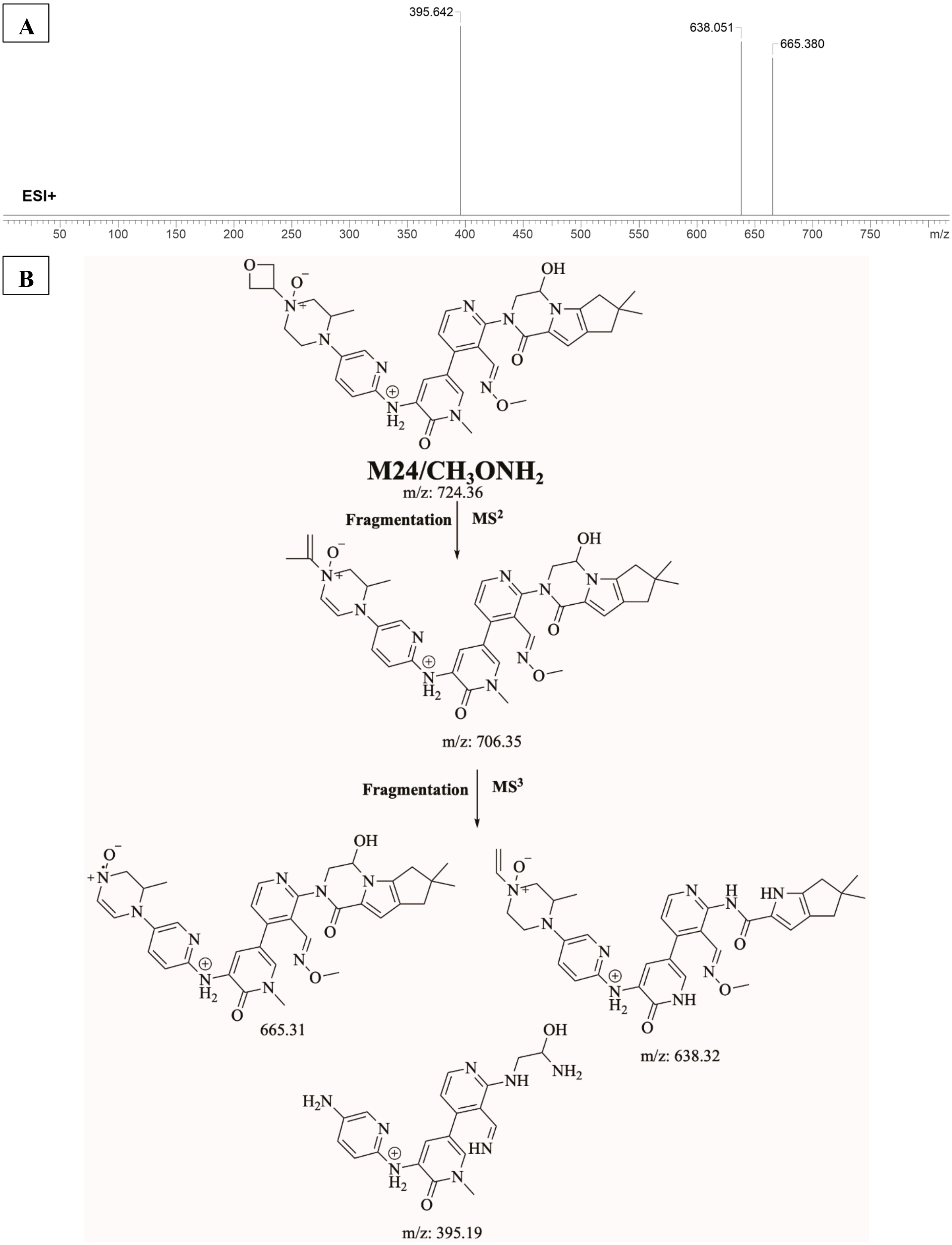

Figure S20. A) Product ion mass spectrum of M24/CH_3ONH_2 methoxylamine adduct, B) Proposed interpretation of fragmentation of M24/CH_3ONH_2.

- **Identification of M25/CH3ONH2 methoxylamine adduct**

M25/CH3ONH2 is proposed to form by the addition of methoxylamine after double hydroxylation of FNB. M25/CH3ONH2 (*m/z* 724) peak appeared at 18.13 minute in fragment ion chromatogram. Dissociation of M25/CH3ONH2 ion at *m/z* 724 inside the collision cell produces one fragment at *m/z* 706. Further investigation using MS^3 analysis of fragment *m/z* 706 yielded five characteristic and qualitative fragment ions at *m/z* 691, *m/z* 593, *m/z* 569, *m/z* 418 and *m/z* 296 (Figure S21).

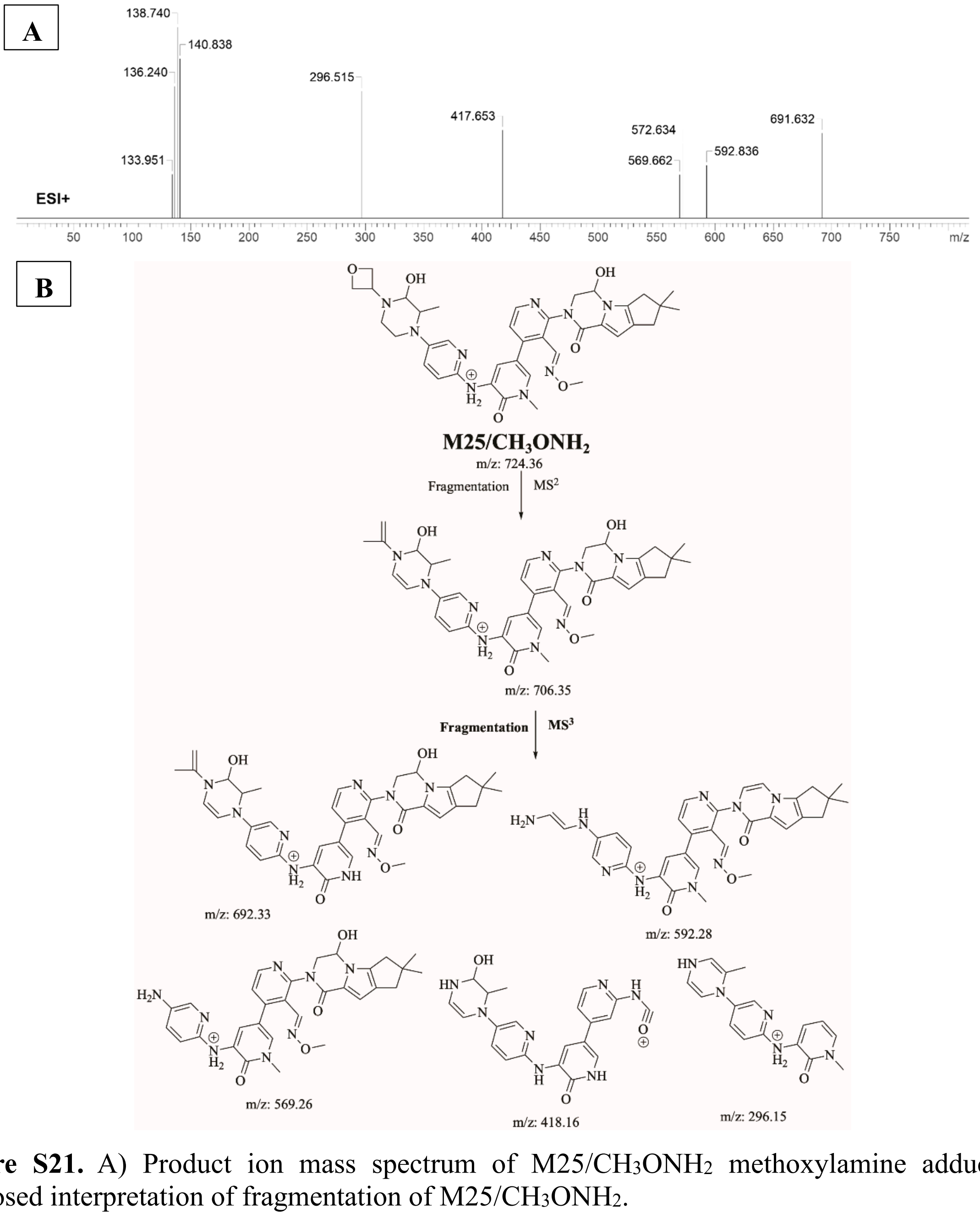

Figure S21. A) Product ion mass spectrum of M25/CH3ONH2 methoxylamine adduct, B) Proposed interpretation of fragmentation of M25/CH3ONH2.

Table S4. Summary of proposed methoxylamine adducts of FNB.

	m/z	Fragments	Retention time	Proposed metabolic reaction
M20/CH_3ONH_2	692	610, 474	22.42	Methoxylamine adduct of FNB.
M21/CH_3ONH_2	521	456, 283	17.01	N-dealkylation and hydroxylation.
M22/CH_3ONH_2	636	522, 361, 358	22.92	N-dealkylation.
M23/CH_3ONH_2	708	616, 606, 565, 335, 240	22.99	Oxidation and opening of piperazine ring.
M24/CH_3ONH_2	724	665, 638, 395	17.6	N-oxidation and hydroxylation.
M25/CH_3ONH_2	724	692, 592, 569, 418, 296	18.13	Double hydroxylation.
